# Diversity and Geographic Distribution of Microsymbionts Associated With Invasive *Mimosa* Species in Southern China

**DOI:** 10.3389/fmicb.2020.563389

**Published:** 2020-10-28

**Authors:** Xiaoyun Liu, Shenghao You, Huajie Liu, Baojuan Yuan, Haoyu Wang, Euan K. James, Fang Wang, Weidong Cao, Zhong Kuan Liu

**Affiliations:** ^1^Key Laboratory of Microbial Diversity Research and Application of Hebei Province, College of Life Science/Institute of Life Science and Green Development, Hebei University, Baoding, China; ^2^The James Hutton Institute, Invergowrie, Dundee, United Kingdom; ^3^Key Laboratory of State Forestry Administration for Biodiversity Conservation in Southwest China, Southwest Forestry University, Kunming, China; ^4^Institute of Agricultural Resources and Regional Planning of CAAS, Beijing, China; ^5^Institute of Agro-resources and Environment, Hebei Academy of Agriculture and Forestry Sciences, Shijiazhuang, China

**Keywords:** ecological distribution, *Mimosa*, phylogenetic diversity, rhizobia, soil conditions

## Abstract

In order to investigated diversity and geographic distribitution of rhizobia associated with invasive *Mimosa* species, *Mimosa* nodules and soils around the plants were sampled from five provinces in southern China. In total, 361 isolates were obtained from *Mimosa pudica* and *Mimosa diplotricha* in 25 locations. A multi-locus sequence analysis (MLSA) including 16S rRNA, *atpD*, *dnaK*, *glnA*, *gyrB*, and *recA* identified the isolates into eight genospecies corresponding to *Paraburkhleria mimosarum*, *Paraburkholderia phymatum*, *Paraburkholeria carbensis*, *Cupriavidus taiwanensis*, *Cupriavidus* sp., *Rhizobium altiplani*, *Rhizobium mesoamericanum*, and *Rhizobium etli*. The majority of the isolates were *Cupriavidus* (62.6%), followed by *Paraburkholderia* (33.5%) and *Rhizobium* (2.9%). *Cupriavidus* strains were more predominant in nodules of *M. diplotricha* (76.2) than in *M. pudica* (59.9%), and the distribution of *P. phymatum* in those two plant species was reverse (3.4:18.2%). Four symbiotypes were defined among the isolates based upon the phylogeny of *nodA*-*nifH* genes, represented by *P. mimosarum*, *P. phymatum*–*P. caribensis*, *Cupriavidus* spp., and *Rhizobium* spp. The species affiliation and the symbiotype division among the isolates demonstrated the multiple origins of *Mimosa* rhizobia in China: most were similar to those found in the original centers of *Mimosa* plants, but *Cupriavidus* sp. might have a local origin. The unbalanced distribution of symbionts between the two *Mimosa* species might be related to the soil pH, organic matter and available nitrogen; *Cupriavidus* spp. generally dominated most of the soils colonized by *Mimosa* in this study, but it had a particular preference for neutral-alkaline soils with low fertility whereas. While *Paraburkholderia* spp. preferred more acidic and fertile soils. The *Rhizobium* spp. tended to prefer neutral–acidic soils with high fertility soils.

## Introduction

Leguminous plants are important for their ability to fix-nitrogen in symbiosis with rhizobia, which makes them critical ecologically and economically. Ecologically, the symbiotic *N*-fixation not only supply the *N* nutrition to the host legume, but also enhance the soil *N* content by its root and shoot remnants (Wang et al., 2019). In addition, the wide distribution and specificity between the legume species and their microsymbionts make the same legume species form symbiosis with distinct rhizobial populations/species in the different geographic regions. Therefore, the growth of a legume plant in a certain region could enrich its corresponding rhizobia adapted to the local environment, e.g., the rhizobia are selected by both the host legumes and the soil conditions, mainly soil pH, nutrient (*N*, *P*, *K* and organic material) contents, and salinity (Wang et al., 2019). With the mentioned concern, characterization rhizobia associated with the same legume species grown in different regions will help to understand the evolution or diversification of rhizobia under the double selection from both the host plant and the soil condition, as well as help for screening the high effective rhizobial strains in agricultural sustainable development.

*Mimosa* species are able to form nitrogen fixing symbiotic associations with soil bacteria collectively termed “rhizobia” (Sprent, 2009; Sprent et al., 2017). Currently, rhizobia are found in two classes: Alpha-rhizobia including species in the well-known genus *Rhizobium* and other genera in the class Alphaproteobacteria, and Beta-rhizobia covering the symbiotic species in genera *Paraburkholderia* (splited from *Burkholderia*), *Cupriavidus* and *Trinickia symbiotica* in the class Betaproteobacteria (Gyaneshwar et al., 2011; Peix et al., 2015; Beukes et al., 2017; Sprent et al., 2017; Los Santos et al., 2018). Species in *Mimosa* genus and another large mimosoid genus *Calliandra* mainly nodulate with Beta-rhizobia, particularly *Paraburkholderia* and *Trinickia*, in its native range in South America, suggesting that the two partners co-evolved (Chen et al., 2005a; Bontemps et al., 2010; dos Reis et al., 2010; Los Santos et al., 2018; Silva et al., 2018). In addition, the three main invasive *Mimosa* species (*M. diplotricha*, *C. Wright*, *M. pigra* L., and *M. pudica* L., originated from the netotropics) in Assia, Australia and the Pacific region also preferred Beta-rhizobia for nodulation (Chen et al., 2001, 2003a,b, 2005b; Liu et al., 2007, 2011, 2012; Parker et al., 2007; Elliott et al., 2009; Andrus et al., 2012; Klonowska et al., 2012; Gehlot et al., 2013; Melkonian et al., 2014), and they are closely related to the microsymbionts of *Mimosa* and related genera in their original regions (Bournaud et al., 2013; da Silva et al., 2012; Mishra et al., 2012; Taulé et al., 2012; Platero et al., 2016; de Castro Pires et al., 2018). For exotic nodulating legumes, access to compatible rhizobial strains in new environments is a critical factor for their successful establishment, and hence, their ability to survive and spread will depend on the presence of compatible symbionts in the soil (Parker et al., 2007). Several studies have indicated that invasive legumes, such as *Mimosa* species and *Dipogon lignosus*, have been introduced into their invasive environments together with their symbionts (Liu et al., 2014). Although Alpha-rhizobia have occasionally been isolated from *Mimosa* species in South American, they either failed to nodulate their hosts of origin or did so ineffectively (Barrett and Parker, 2006; Elliott et al., 2009; Klonowska et al., 2012; Mishra et al., 2012). In addition, Alpha-rhizobia (*Rhizobium* or *Ensifer* species) appear to be the dominant symbionts of native *Mimosa* spp. in central Mexico, central Brazil and India, where the soils presented neutral –alkaline pH values (Wang et al., 1999; Gehlot et al., 2013; Baraúna et al., 2016; de Castro Pires et al., 2018). These discrepancies in symbiont preference between *Mimosa* species in different regions might be attributed to the soil characteristics, particularly pH, as the Beta-rhizobia, are highly tolerant to the low fertility acidic soils (dos Reis et al., 2010; de Castro Pires et al., 2018).

The herbaceous perennial legume *Mimosa pudica* was first introduced into Taiwan Province of China in 1645 as an ornamental plant (Wu et al., 2003) and it has been dispersed throughout the tropical and subtropical China. It is a plant serving as valuable bio-resource for various uses, such as green manure, fodder crops, honey source, as well as a medicine used in zoster therapy (Wang, 2014) and treating kidney disease. However, this naturalized plant is highly invasive causing considerable ecological damage e.g., by affecting the growth of grass lawns and as a common exotic weed in rice paddy fields (Guan et al., 2006). Therefore, *M. pudica* and *M. diplotricha* another invasive plant without history record, are considered to be serious pests widely dispersed in waste-grounds and city suburbs of China. It has been estimatied that the access to compatible rhizobial strains in new environments is a critical factor for the successful establishment of exotic nodulating legumes (Parker et al., 2007). For getting the compatible symbionts in the introduced region. The invasive legumes, such as *Mimosa* species (see above) and the invasive papilionoid legume *Dipogon lignosus* (L.) Verdc., may have been introduced into their invasive environments together with the symbionts from their original region (Liu et al., 2014). Or they may form the symbiosis with rhizobia adapted to the local environment and adopted the corresponding symbiotic genes through lateral gene transfer, like the cases of chickpea (*Cicer arietinum* L.) rhizobia in China (Zhang et al., 2017, 2018). Previously, *Burkholderia* spp. and *Cupariavidus taiwanensis* have been isolated from *Mimosa* species grown in China (Chen et al., 2001, 2005a; Liu et al., 2007, 2011, 2012). In which *C. taiwanensis* was recognized as native to Taiwan and the *Burkholderia* spp. were estimated as rhizobia introduced together with the host plants (Chen et al., 2005a). Furthermore, preference for *Cupriavidus* by *M. pudica* and *Burkkholderia* by *M. pigra* in Taiwan (Chen et al., 2005b), while *Cupriavidus* by *M. diplotricha* and *Burkkholeria* by *M. pudica* in Yunnan (Liu et al., 2012) demonstrated that both the host plants and the geographic regions affected the symbiosis combination between the legume and the rhizobia in the introduced regions. However, no soil conditions were considered in these previous studies about the *Mimosa* rhizobia in China.

In order to explore how the environmental factors and the host species influenced the composition and competitiveness of *Mimosa* symbionts, we performed this study to investigate the *Mimosa* symbionts in in different geographic regions for evaluating the competitiveness of different rhizobial species associated with invasive *Mimosa* spp. under varied soil traits (organic matter, *N*, *P*, *K*, and pH).

## Materials and Methods

### Isolates and Strains

**FIGURE 1:**
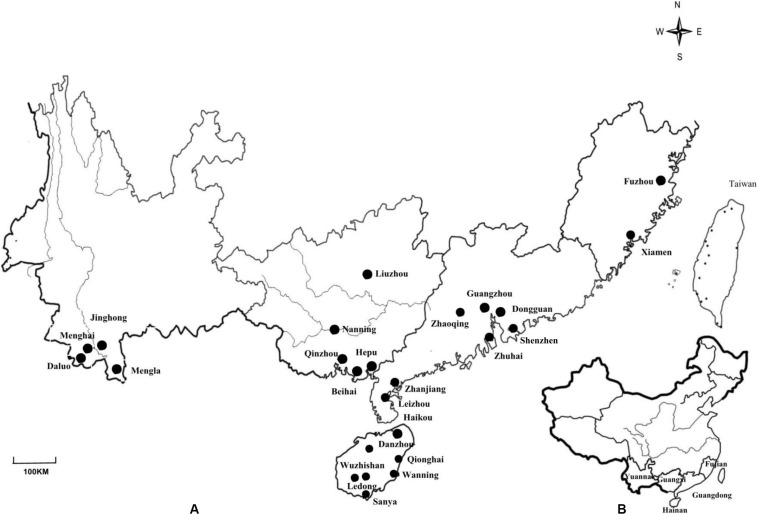
**(A)** Map of Yunnan, Guangxi, Guangdong, Fujian and Hainan Provinces in China showing the locations of the 25 sites where nodules from invasive *Mimosa* species were sampled. **(B)** Map showing the location of the sampled provinces in China.

Root nodules were sampled from *M. pudica* and two varieties of *M. diplotricha* var. inermis (Adelbert) Verdcourt. and var. diplotricha, growing in 25 locations of five Chinese provinces in the subtropical and tropic regions (Figure 1), as described previously (Liu et al., 2007). Root nodules were collected from three plant individuals at each site and were stored over silica gel in closed vials until their isolation in the laboratory (Vincent, 1970). Root nodule bacteria were isolated and purified from the nodules on yeast mannitol agar (YMA) using the standard procedure (Vincent, 1970). The nodule isolates obtained in this study were maintained in yeast mannitol broth (YMB) supplied with 20% (v/v) glycerol at −80°C.

### Molecular Typing of Rhizobia

For grouping the isolates by genomic analysis, total DNA of each isolate and the reference strains *Paraburkholderia mimosarum* LMG23256^T^, *Paraburkholderia phymatum* LMG21445^T^, *Paraburkholderia caribensis* LMG18531^T^, *Cupriavidus taiwanensis* LMG19424^T^, *Cupriavidus* sp. SWF66294 (Liu et al., 2011) was extracted from 5 mL of culture in YMB (Vincent, 1970). The extracted genomic DNA was used as template DNA for BOX-AIR and the PCR-based RFLP (restriction fragment length polymorphism) of 16S rRNA gene (rDNA). The rDNA primers were fD1 (5′-AGAGTTTGATCCTGGCTCAGA-3′) and rD1 (5′-AAGGAGGTGATCCAGCC-3′) (Weisburg et al., 1991). The BOXAIR primer 5′-CTA CGG CAA GGC GAC GCT GAC G-3′ (Versalovic et al., 1991) was used for BOX-PCR. Both PCRs were carried out in a total volume of 25 μL of the reaction mixture with the PCR procedure of Nick et al. (1999) and the products were checked by electrophoresis in 1% (w/v) of a garose gel.

For analysis of RFLP, aliquot (5–10 μL, depending on the concentration) of PCR products was digested separately with the restriction endonucleases *Hae III* (GG∣CC), *Rsa I* (GT∣AC), *Hif I* (G∣ANTC), and *Msp I* (C∣CGG) (Laguerre et al., 1994) as specified by the manufacturer with an excess of enzyme (5 U per reaction). The restriction fragments were separated by horizontal electrophoresis in agarose (2%, w/v) gels (14 cm in length) at 80 V for 3 h and were visualized by staining with ethidium bromide. Strains or isolates with different RFLP patterns were designated into distinct rDNA types. The BOX-AIR products were separated by electrophoresis in 1.5% (w/v) agarose gels containing ethidium bromide and were photographed under UV light. The BOX profiles were distinguished by their different band patterns, e.g., the isolates sharing the same pattern were designed as the same BOX pattern.

### Characterization of Whole Cell Protein by SDS-PAGE

Bacterial strains were grown until the end of the exponential phase at 28°C for 2 days on YMA. The cells were collected and washed twice in 10 mM Tris–HC1, pH 7.6, the pellet obtained by centrifugation (5000 × *g*, 10 min at 4°C) was weighed and the cells were resuspended in 10 mM Tris–HC1 to a concentration of 10 mg ml^–1^ using. Then, the same volume of 2 × treatment buffer (0.5 g of SDS, 3 ml of glycerol, 1 ml of 2-mercaptoethanol, 4 mg of bromophenol blue, 2 ml of 1 M Tris-hydrochloride, and distilled water to make a final volume of 10 ml at pH 6.8) was added. The samples were incubated at 100°C for 20 min and immediately stored at −20°C after cooled on ice. The SDS-polyacrylamide gel (200 mm × 200 mm × 1 mm) were used for electrophoresis according to Laemmli (1970). The samples were incubated at 100°C for 10 min before the sample loading. Twenty-five samples per gel were subjected to the discontinuous slab gel electrophoresis at 250 V in an SDS-Tris-glycine buffer system, as described by Laemmli (1970). The protein patterns were visualized by silver staining (Tan et al., 1997). The protein bands were scanned with a Densitometer Extra-Scanner and strains sharing the identical band patterns were designed into the same SDS-PAGE pattern.

### Phylogenetic Analyses of Housekeeping Genes and Symbiotic Genes

The 16S rRNA amplified by PCR as described above was purified and sequenced directly (Weisburg et al., 1991) commercially in the Beijing Genomics Institute (BGI). The sequences acquired in this study were aligned with related sequences extracted from GenBank using Clustal W (Thompson et al., 1997). Maximum likelihood phylogenetic trees were constructed and were bootstrapped with 1000 pesudo-replicates using Mega 6.1 (Tamura et al., 2013).

Multilocus sequence analysis (MLSA) based on the five housekeeping genes *atpD* (encoding for the ATP synthase beta-chain), *recA* (recombinase A), *dnaK* (DnaK chaperone), *gyrB* (DNA gyrase, beta-subunit), and *glnA* (glutamine synthetase I) widely used to differentiate rhizobial species (Vinuesa et al., 2005; Martens et al., 2007, 2008) was also employed in the present study. The five genes were independently amplified using corresponding primer pairs reported in previous studies (Payne et al., 2005; Vinuesa et al., 2005; Andam and Parker, 2007; Martens et al., 2008), or designed in this study (Supplementary Table S1). The PCR products were checked by electrophoresis in 1% (w/v) agarose gel. After purified with the Solarbio DNA purification kit (Beijing Solarbio Science and Technology Co., Ltd.), the amplicons were sequenced directly using the same primers in BGI mentioned above. The separated sequences of *recA* and the combined sequences of *atpD*, *glnA*, *gyrB*, and *dnaK*, and their combined sequences were aligned using Clustal W with those from type strains of the defined bacterial species (obtained from the NCBI database). Distance calculation and construction of the gene phylograms were performed using the Maximum likelihood method and the bootstrapping algorithms with 1000 pseudo-replicates were carried out in MEGA 6.0 (Tamura et al., 2013). Phylogenies were also constructed using the concatenated sequences of 16S rRNA and the five housekeeping genes by Maximum likelihood method.

Fragments of the symbiosis genes *nifH* and *nodA* genes were amplified and sequenced using primers reported previously (Haukka et al., 1998; Laguerre et al., 2001; Liu et al., 2012) as well as with the new primers designed in this study (Supplementary Table S1). The visualization purification and sequencing of the *nifH* and *nodA* amplicons were performed same as that mentioned for the housekeeping genes. The sequences were deposited in the NCBI database and were used for alignment and construction of the phylogenies using the same methods described above for the 16S rRNA gene.

The obtained nucleic acid sequences were submitted in GENEBANK, and the accession numbers in this paper was MT337483 as list in Supplementary Table S2.

### Nodulation Tests

A total of 98 representative strains were used in the nodulation tests that were selected according to their affiliations of genotypes based on the results of 16S rRNA sequencing, protein patterns in SDS-PAGE, and genomic fingerprinting by BOX-PCR. *M. pudica* seeds were scarified using concentrated sulfuric acid for 10 min, rinsed several times with sterile water, and then surface-sterilized in 3.2% (w/v) sodium hypochlorite followed by several rinses with sterile water. They were then placed on 0.8% water-agar at 4°C for 3 days, and after germinated at 28°C until the seedlings developed roots of 0.5–1 cm in length. Two seedlings then were transplanted into a sterile glass tubes (30 cm × 200 cm) with nitrogen-free plant nutrient solution (Vincent, 1970) in 0.8% agar. The seedlings were then inoculated separately with 0.1 mL liquid cultures of each test strain (about 10^8^ cells mL^–1^). Five replicates were used and controls without inoculation were included. The plants were placed in a growth cabinet under conditions described previously (Zhang et al., 2012). The representative strains *Paraburkholderia* spp. SWF66044, SWF66029, and *C. taiwanensis* SWF66166, SWF66194, and SWF66322 from Liu et al. (2012) were also used for cross-inoculation tests with ten other leguminous species: *Glycine max* (Linn.) Merr., *Pisum sativum* L., *Galega officinalis* L., *Phaseolus vulgaris* Linn., *Vigna unguiculata* L. Walp, *Lotus corniculatus* L., *Medicago sativa* L., *Trifolium repens* L., *Macroptilium atropurpureum* (Moc. and Sessé ex DC.) Urb. and *Leucaena leucocephala* (Lam.) de Wit. Seed treatments and inoculation details were the same as described above. Plants were checked for nodule formation at 35 d after inoculation.

### Correlation Between Soil Types and Distribution of Rhizobial Groups

In order to evaluate the influence of soil characters on the symbiosis between *Mimosa* spp. and different rhizobial types, soil, and root nodules were sampled intensively from 13 locations (59 sites) including Hepu, Beihai, and Nanning city in Guangxi (GXh,GXb, and GXn), Zhangjiang, Leizhou, Mazhang, and Foshan town in Guangdong (Gzj, Gl, Gm, and Gf), Jinhong and Mangshi town in Yunan (Yj and Ym) and Ledong, Wuzhishan, Wanning and Danzhou in Hainan (Hl, Hw, Hwn, and Hd), which were main districts for *Mimosa* speies and habitats for diverse rhizobia. For most locations, four or more sites with minimum distance of 5 km between them were samples, except the location Mangshi town in Yunnan where rhizobial strains were isolated from only one sampling site. Soils were sampled compositely from the root zone of nodule sampled plants (5–20 cm in depth). The soil samples were dried and milled until they could pass through an 80-mesh sieve. Soil alkali-hydrolysable N, available P (using Bray’s hydrochloric acid fluoride ammonium by extraction method), and available K (by ammonium acetate extraction plus flame photometry) were determined with the standard procedures (Du and Gao, 2006). Soil pH was measured using a pH meter (Mettler Toledo) by suspending 5 g soil in 5 mL of distilled water, and organic matter was measured using the potassium dichromate volumetric method (Du and Gao, 2006). Rhizobial isolation, and genus/rRNA type identification by PCR-based RFLP of 16S rRNA gene were performed same as mentioned above.

Based on the soil characters, the soil samples in the 59 sites were sorted into soil types by SPSS 13.0 (SPSS Inc., Chicago, IL, United States), in terms of their pH values and the nutritional characteristics, including organic matter (OM), alkali-hydrolysable N, available P, and available K.

The data was standardized, then using construct UPGMA dendrogram (Sneath and Sokal, 1973) for soil clustering. Principal component analysis (PCA) on a correlation matrix was used to evaluate the distribution of the different rhizobial rRNA types in the 59 sites to see if they correlated with the soil characteristics. Data analysis and graphs were performed using Past 3.0.

## Results

### Isolation and Genotyping of the Rhizobia

In total, 361 strains were isolated from the nodules of *M. pudica* and *M. diplotricha* sampled in the 25 locations in southern China (Figure 1 and Table 1). The majority of the isolates were obtained from *M. pudica* (83.7%), and minor from *M. diplotricha* (16.3%), which most likely reflects the relative abundance of these two plant species in the sampling sites. By 16S rRNA PCR-RFLP analysis, six rRNA types were revealed (Table 1), which were recognized as members of *Paraburkholderia* (three rRNA types with 55, 57, and 9 strains; 33.5%), *Cupriavidus* (two rRNA of types with 98 and 128 strains; 62.6%) and *Rhizobium* (a single rRNA type with 9 strains; 3.9%). *Paraburkholderia* genotypes I and II, *Cupriavidus* genotypes I and II, as well as *Rhizobium* genotype were isolated from both *M. pudica* and *M. diplotricha*; while. *Paraburkholderia* genotype III was only isolated from *M. pudica*.

**TABLE 1:** Occurrence of rhizobial genotypes isolated from two invasive *Mimosa* species in southern China.

Strains	Numbers*	Host	SDS-PAGE patterns	BOX-PCR patterns	Sampling location
***Paraburkholderia* genotype I**	55		18	10	13
*P. mimosarum* LMG23256^T^		*M. pigra*	ND	ND	Taiwan
HBU52030 and 20 other strains	21	*M. pudica*	P9–P13	R3, R5, R8, R9	Gd, Gl, Gs, Gzh, Gzj
HBU53012 and 9 other strains	10	*M. pudica*	P4–P8	R4–R7	GXn, GXh, GXq
HBU08184 and 9 other strains	10	*M. pudica*	P1, P2, P3	R1–R3	Hwn
HBU67638, HBU67639	2	*M. pudica*	P16	R6	Yml
BHU36005, HBU36004	2	*M. pudica*	P15	R4	Fx
HBU08166 and 5 other strains	6	*M. diplotricha*	P14, P17	R4, R10	Hwn
HBU67640 and 3 other strains	4	*M. diplotricha*	P10, P18	R5, R6	Yj, Ymh
***Paraburkholderia* genotype II**	57		11	6	9
*P. phymatum* LMG21445^T^		*M. pudica*	ND	ND	French Guiana
HBU52006 and 36 other strains	37	*M. pudica*	P20, P22, P24–P27, P24–P27	R11, R12, R15R13, R16	Gl, Gzh, Gzj, Gs
HBU52001 and 13 other strains	14	*M. pudica*	P19–P23	R11–R14	GXl, GXh, GXn
HBU67642, HBU67643	2	*M. diplotricha*	P28	R15	Yd
HBU35004 and 3 other strains	4	*M. pudicaa*	P29	R17	Ff
***Paraburkholderia* Genotype III**	9		3	3	3
*P. caribensis* LMG 18531^T^		Soil	ND	ND	Martinique
HBU510005 and 4 other strains	5	*M. pudica*	P29, P30	R17, R18	Gs, Gzh
HBU54006 and 3 other strains	4	*M. pudica*	P31	R19	GXl
***Cupriavidus* Genotype I**	98	*M. pudica*	27	16	13
*C. taiwanensis* LMG19424^T^		*M. pudica*	ND	ND	Taiwan
HBU52045 and 42 other strains	43	*M. pudica*	P42, P46–P51	R27, R29, R30, R31	Gd, Gs, Gzh
HBU53027 and 7 other strains	8	*M. pudica*	P43, P44, P45	R26–R28	GXb, GXh, GXn
HBU08065 and 34 other strains	35	*M. pudica*	P30–P42	R20–R26	Hd, Hh, Hl, Hwn, Hwz
HBU36003	1	*M. pudica*	P52	R32	Fx
HBU52008	1	*M. diplotricha*	P53	R30	Gl
HBU08093 and 8 other strains	9	*M. diplotricha*	P54, P55, P56	R30, R33–R35	Hwn, Hwz
HBU67648	1	*M. diplotricha*	P56	R35	Yj
***Cupriavidus* genotype II**	128		31	21	15
*Cupriavidus* sp. SWE66294		*M. pudica*			Jinhong of Yunnan
HBU52009 and 50 other strains	51	*M. pudica*	P66–P76	R46–R50	Gd, Gl, Gs, Gqz, Gzj, Gzq
HBU53034 and 8 other strains	9	*M. pudica*	P66	R45	GXb
HBU08001 and 31 other strains	32	*M. pudica*	P57–P65	R36–R44	Hh, Hl, Hq, Hwn, Hwz, Hs
HBU36001 HBU36002	2	*M. pudica*	P77	R47	Fx
HBU52018 and 8 other strains	9	*M. diplotricha*	P78–P80, P85	R45, R50, R55	Gl, Gzq, Gzj
HBU08084 and 18 other strains	19	*M. diplotricha*	P74, P79, P81–P84	R45, R51–R54	Hd, Hl, Hwz
HBU67645 and 5 other strains	6	*M. diplotricha*	P83, P84, P86, P87	R53, R54, R56	Yd, Yml, Yj
***Rhizobium* sp. Genotype**	14		7	7	6
HBU08051 and 9 other strains	10	*M. pudica*	P88–P92	R57–R61	Hd, Hl, Hwn, Hwz
HBU53006, HBU53007	2	*M. pudica*	P93	R62	GXq
HBU08133, HBU08134	2	*M. diplotricha*	P94	R63	Hd

*Numbers refers to strains isolated from different provinces, different plants, and total of one genotype/total of sampling locations, the 7 sampling locations of Gd, Gl, Gs, Gzj, Gzh, Gzq, and Ggz refers to Dongguan, Leizhou, Shenzhen, Zhanjiang, Zhuhai, Zhaoqin and Guangzhou of Guangdong province; five sampling locations of GXb, GXh, GXn, GXl, and GXq refers to Beihai, Hepu, Nanning, Liuzhou, and Qinzhou of Guangxi province; 7 sampling locations of Hd, Hh, Hl, Hq, Hwn, Hwz, and Hs refers to Danzhou, Haikou, Ledong, Qionghai, Wanning, Wuzhishan, and Sanye of Hainan province; four sampling locations of Yd, Yj, Ymh, and Yml refers to Daluo, Jinhong, Menghai and Mengla of unnan province and two sampling locations of Ff and Fx refers to Fuzhou and Xiaomen of Fujian province.

The multivariate statistical analysis (Supplementary Table S4) for conducting population distribution of the six genotypes associated with *M. pudica* and *M. diplotricha* in the four provinces (Guangdong, Guangxi, Hainan, Yunnan, and Yunnan data also from previous study in Liu et al., 2012) of China showed no significant difference (*p* = 0.094, >0.05), but it was significantly different (*p* = 0.039, <0.05) for distribution of the three genera *Paraburkholderia*, *Cupriavidus*, and *Rhizobium* in the four provinces, for example, the *Rhizobium* isolates were mainly from Hainan.

### Fingerprinting of the Isolates for Estimation of Genetic Diversity

In analyses of genetic diversity, a total of 97 protein profiles and 51 BOX PCR profiles were distinguished among the 361 strains (Table 1), revealing a high level of diversity among them. *Paraburkholderia* rRNA type I contains 18 protein profiles and 10 BOX PCR profiles; *Paraburkholderia* rRNA type II contains 11 protein profiles and 6 BOX PCR profiles; and *Paraburkholderia* rRNA type III contains 3 protein profiles and 3 BOX PCR profiles; *Cupriavidus* rRNA type I contains 27 protein profiles and 16 BOX PCR profiles; and *Cupriavidus* rRNA type II contains 31 protein profiles and 9 BOX PCR profiles. *Rhizobium* rRNA type contains 7 protein profiles and 7 BOX PCR profiles. The greater number of patterns in the *Cupriavidus* populations suggested that they were more diverse than the *Paraburkholderia* populations.

### Phylogenies by MLSA and Affiliation of the Isolates

**FIGURE 2:**
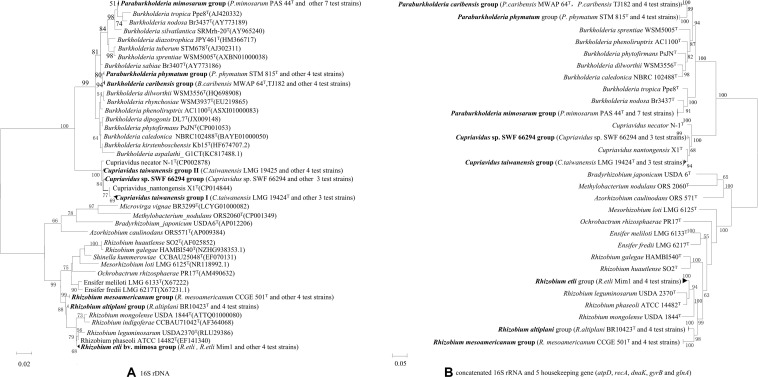
Phylogenies of 16S rRNA gene and the concatenated 16S rRNA and five housekeeping genes (*atpD*, *recA*, *dnaK*, *gyrB*, and *glnA*) in rhizobial strains isolated from nodules on invasive *Mimosa* spp. in southern China. The Maximum likelihood phylogenies are based on **(A)** 16S rRNA gene sequences (1320 bp); **(B)** concatenated 16S rRNA and five housekeeping gene sequences (3706 bp).

Out of the total collection, 34 strains (Supplementary Table S2) were selected according to their different rRNA types, protein patterns, BOX profiles, host species and collected sites for full 16S rRNA and housekeeping gene sequencing. The relationships of the *Mimosa* isolates were high similar in 16S rRNA gene phylogeny (Supplementary Figure S2) and in the MLSA-based phylogeny deduced from the concatenated sequences of 16S rRNA and the five housekeeping genes (Figure 2B). Five groups were defined among the beta-rhizobial isolates at the species level (similarities ≥96.4%), which corresponded to (1) *P. mimosarum* (*Paraburkholderia* rRNA type I), (2) *P. phymatum* (*Paraburkholderia* rRNA type II), (3) *P. caribensis* (*Paraburkholderia* rRNA type III), (4) *C. taiwanensis* (*Cupriavidus* rRNA types I), and (5) *Cupriavidus* sp. SWF66294 (*Cupriavidus* rRNA types II). The *Rhizobium* isolates obtained in this study were grouped into three species corresponding to *R. etli*, *R. mesoamericanum*, and *R. altiplani*. The phylogenies of the individual housekeeping genes (*atpD*, *recA*, *dnaK*, *gyrB*, and *glnA*) (Supplementary Figures S1–S5) were generally consistent with that of 16S rRNA gene and the MLSA (Figure 2), except the isolates of *R. etli* that was a unique linage separated from all the defined species in *atpD* phylogenetic tree (Supplementary Figure S3).

### Sequencing and Phylogenetic Analysis of Symbiosis Genes

**FIGURE 3:**
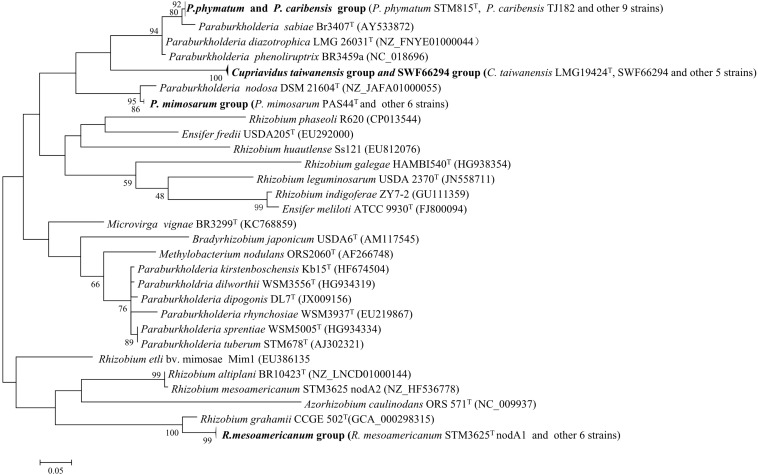
Phylogenetic tree based on *nodA* gene sequences (230 bp) showing the groups of the rhizobial strains isolated from *Mimosa* spp. The tree was constructed by using Maximum likelihood method. Bootstrap values are indicated (>50) in the main nodes in a bootstrap analysis of 1,000 replicates, 5% substitutions per site.

This analysis were performed for the 34 representative strains mentioned above. Four *nodA* and four *nifH* lineages were defined among them (Figure 3 and Supplementary Figure S6). The phylogenies of the *nodA* and *nifH* genes of the isolates were the same and they were incongruent in several cases with that of the housekeeping genes, e.g., both the Alpha-and Beta-rhizobia formed two clades and they were intermingled. *P. caribensis* strains and *P. phymatum* strains shared similar symbiosis genes, while *P. mimosarum* presented another lineage (Figure 3 and Supplementary Figure S6). The strains in both *Cupriavidus* genotypes I and II, including *C. taiwanensis* LMG19424^T^, formed the third lineage in the symbiosis gene phylogeny, which was inserted between the two lineages of the *Paraburkholderia* species. All the three *Rhizobium* species identified in the present study shared the same symbiosis gene and formed the forth lineage represented by that of *R. mesoamericanum* STM3625 isolated from *M. pudica* in Mexico.

### Nodulation Test

All 98 representative strains selected from different groups according to their patterns in 16S rRNA PCR-RFLP, SDS-PAGE protein and BOX-PCR profiles (Table 1 and Liu et al., 2012) formed nodules on *M. pudica*. Cross-inoculation studies showed that only the invasive mimosoid legume, *L*. *leucocephala* could nodulate with all five test strains (*Paraburkholderia* spp. SWF66044, SWF66029, *C. taiwanensis* SWF66166, SWF66194, and SWF66322), which is in keeping with its neotropical origin, close relatedness to *Mimosa*, and its known promiscuity.

### Correlation Between Sampling Sites and the Distribution of *Mimosa* Symbionts

Soil characteristics in the 59 sample sites at 13 locations are presented in Supplementary Material (Supplementary Table S3). Briefly, among these sites, the soils pH ranged from 5.2 to 7.78; soil organic matter ranged from 3.51 to 43.82 mg kg^–1^; alkali-hydrolysable N ranged from 1.49 to 82.16 mg kg^–1^; available P ranged from 0.94 to 88.98 mg kg^–1^; and available *K* ranged from 4.34 to 321.84 mg kg^–1^ (Supplementary Table S3).

**FIGURE 4:**
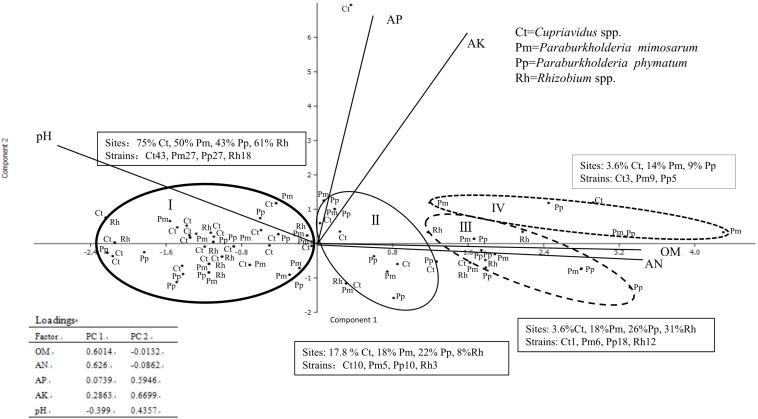
Comparison of the distribution of four communities of rhizobial types associated with invasive *Mimosa* species in southern China by principal component analysis of total N, available K, available P, precipitation and pH. The principal component one revealed correlations of available K with available P and pH. The principal component two is characterized by precipitation and total N and organic matter. Ellipses represent 90% confidence limits. The word in text box means each occupied sites percent and species constitute in different soil types.

The locations and the soil parameters within each sampling site were analyzed via cluster analysis by SPSS and PCA, which revealed main four groups with distinct soil patterns, an unusual soil site as another type for *Cupriavidus* spp. surviving (because it is distant from other soil point) was not considered (Figure 4). We obtained 200 rhizobial strains from the 59 sites and they were identified into four groups as *Cupriavidus* spp., *P. mimosarum*, *P. phymatum*, and *Rhizobium* spp., and each group grown soil nutrition and pH range displayed as Table 2. In PCA, only the existence (not abundance) of the different group in each site was considered, and they were in relation to their spatial distribution (Figure 4 and Table 2). The soil PCA resulted in three components with eigenvalues greater than one which explained 75.3% of the total variance (first component: 47.7%, second component: 27.6%, third component: 14.2%). However, the third component did not provide any further information above the first two components, and hence was excluded from the interpretation. The first component revealed the positive correlation of OM and available *N*, and a contrasting correlation of these with pH (loading factors = 0.58, 0.60, and 0.37, respectively) with soil types I, II, III, and IV; the second component is characterized by a positive correlation of available *P* and available *K* (loading factors = 0.55, 0.73). On the PCA scatter plot, the four soil patterns are almost completely separated.

**TABLE 2:** Characteristics of the habitats of four rhizobial groups from invasive *Mimosa* species in southern China.

Group	Organic matter (mg/kg)			Available N (mg/kg)			Available P (mg/kg)			Available K (mg/kg)			Soil pH		
	Range	Mean	SD	Range	Mean	SD	Range	Mean	SD	Range	Mean	SD	Range	Mean	SD
*Cupriavidus* spp.	3.56–43.82	13.98	8.24a	1.49–65.73	21.02a	15.6a	1.08–88.98	9.94	21.32a	10.96–321.84	73.46	73.3a	5.67–7.78	6.95	0.48a
*Paraburkholderia mimosarum*	3.51–35.94	17.54	9.63a	4.19–82.16	34.44a	22.49ab	0.94–16.42	6.67	4.82a	10.02–214.5	84.76	57.71a	5.2–7.38	6.19	0.63b
*Paraburkholderia phymatum*	1.95–35.32	16.84	9.91a	1.49–73.2	33.15a	22.42ab	0.94–29.3	5.39	6.70a	4.34–214.5	71.17	61.36a	5.31–7.38	6.35	0.67b
*Rhizobium* spp.	3.51–32.14	15.4	9.72a	1.49–68.67	28.46a	21.71ab	1.52–18.46	6.08	5.42a	16.3–174.74	59.78	39.85a	5.67–7.78	6.53	0.65b

*The data use *F*-test by SPSS17.0, the result showed only soil pH was significant among different groups (*p* = 0.001, ≤0.01), different letters means subset by Turkey test.*

In total, the 59 sampling sites were plotted onto the soil-site PCA (Figure 4), and the rhizobial species that were isolated from each site and isolates numbers are indicated in Supplementary Table S3. Four soil types corresponded to the localization site of the different rhizobial types associated with *Mimosa* in southern China. Soil category I (32 sites, 115 isolates) was the major soil type characterized by low fertility, relatively high available *P*, and neutral-alkaline pH and occupied by the majority of the *Mimosa* symbionts obtained in this study. In this type of soil, the rhizobial community contained 37.4% *Cupriavidus*, 23.5% *P. mimosarum*, 23.5% *P. phymatum* and 15.5% *Rhizobium* spp. strains which covered 75% of the *Cupriavidus* isolates, 50% of the *P. mimosarum*, 43% of the *P. phymatum* and 62% of the *Rhizobium* spp. strains. Soil category II (10 sites, 28 isoates) harbored 35.7% *Cupriavidus*, 17.9% *P. mimosarum*, 35.7% *P. phymatum*, and 10.7% *Rhizobium* spp. strains; it characterized by intermediate fertility and neutral pH. Soil category III (11strains, 37 isoates) tended to acid-neutral pH, with lower fertility and lower available *P* and *K*; it contained 2.7% *Cupriavidus* (1 strain), 16.2% *P. mimosarum*, 48.6% *P. phymatum*, and 32.5% *Rhizobium* spp. strains. Soil category IV (5 sites, 17 isoates) was quite acidic with high fertility, but with low available *P*; it harbored the rhizobial community with 17.6% *Cupriavidus*, 52.9% *P. mimosarum* and 29.5% *P. phymatum* strains.

Within all the 59 sites, *Cupriavidus* strains habitat in 29 soil sites (49.2% of total sites), but occupied 75%, 17.8%, 3.6, and 3.6% of the sites in soil categories I through IV, respectively. *P. mimosarum* strains habitat in 22 soil sites (37.3% of total sites), and scattered on 50, 18, 18, and 14% of the sites in categories I through IV, respectively, *P. phymatum* strains survive in 23 soil sites (about 39% of total sites), dispersed 43, 22, 26, and 14% of the sites in the soil categories I through IV, respectively, *Rhizobium* spp. strains habitat in 13 soil sites (22% of total sites) belonging to the soil categories I through III, appearing in 61, 3, and 12% of the sites, respectively.

## Discussion

### Mimosa-Nodulating Rhizobial Community in Southern China

Based upon the MLSA resules (Figure 2), the six rRNA types of *Mimosa* rhizobia defined by PCR-based RFLP of 16S rRNA gene (Table 1), could be indentified as 8 species: *Paraburkholderia* rRNA type I as *P. mimosarum*, *Paraburkholderia* rRNA type II as *P. phymatum*, *Paraburkholderia* rRNA type III as *P. caribensis*, *Cupriavidus* rRNA types I as *C. taiwanensis*, *Cupriavidus* rRNA types II as *Cupriavidus* sp., *Rhizobium* sp. genotype as *R. etli*, *R. mesoamericanum*, and *R. altiplani*. These identifications might imply that the PCR-RFLP of 16S rRNA is an efficient method to identify the current beta-rhizobial species, but it is unable to differentiate the alpha-rhizobial species, as evidenced in many previous studies (for example, Huo et al., 2019; Li et al., 2019).

Although four of the six beta-rhizobial genotypes/species defined among the symbionts of *Mimosa diplotricha* and *M. pudica* in southern China (Table 1) were commonly associated with *Mimosa* species in both their native and invasive regions. The *Cupriavidus* sp. represented by strains SWF66294 covering 128 isolates was unique in China. The presence of identical *nodA* and *nifH* in *Cupriavidus* sp. and in *C. taiwanensis* might be evidence that lateral transfer of symbiosis gene between these two species has happened, as reported in the *Lotus*-nodulation *Mesorhizobium* species (Bamba et al., 2019). The situation in the three Rhizobium species was similar. In addition, the intermingling of the Alpha-and Beta-rhizobial clades in the phylogenies of symbiosis genes (Figure 3 and Supplementary Figure S6) also demonstrating the lateral transfer of symbiosis gene between these two rhizobial categories, as estimated previously (see review of Andrews et al., 2018). Therefore, new symbionts of *Mimosa* has been evolved in China under the double selection from host plant (for symbiotic gene background) and the soil conditions (for survival in the local sites). Previously Gehlot et al. (2013) estimated that invasive *Mimosa* spp. do not interact with the symbionts of native legumes; however, the *Mimosa* species could get novel symbiosis adapted to their invaded region by lateral transfer of the symbiosis genes from its known rhizobia to the native relatives. *Cupriavidus* sp. may constitute a new species intermediate between *C. taiwanensis* and *C. nantongensis* (Sun et al., 2016), but more analyses are required to clarify its species affiliation, such as DNA-DNA hybridization (DDH) and/or average nucleotide identity (ANI) with the closest type strains.

*Cupriavidus taiwanensis* is a very common symbiont of invasive *Mimosa* species in South East Asia (Chen et al., 2001, 2003b, 2005a; Elliott et al., 2009; Andrus et al., 2012; Liu et al., 2012, 2011; Gehlot et al., 2013), and it could be the dominant symbiont in some locations for *M. diplotricha* and *M. pudica*, but not for *M. pigra* (Chen et al., 2001, 2003b, 2005b; Klonowska et al., 2012). So, different *Mimosa* species may have distinct preferences for their symbionts (Chen et al., 2005a, b; Elliott et al., 2009). Although *Cupriavidus* is less commonly isolated in the native regions of *Mimosa*, *C. taiwanensis* has been isolated from *M. pudica* (Barrett and Parker, 2006; Mishra et al., 2012) and *M. asperata* (Andam et al., 2007) in Americas. Moreover, *Cupriavidus* strains closely related to *C. necator* and *C. pinatubonensis* were the dominant symbionts of native *Mimosa* in Uruguay (Platero et al., 2016). All these results imply that *C. taiwanensis* might originated in the Americans and was introduced to China together with the *Mimosa* plants.

In the present study, *P. mimosarum*, *P. phymatum*, and *P. caribensis* comprised 15.2, 15.8, amd 2.5% of the total isolates from 14, 11, and 3 sample sites, respecitively, *P. mimosarum* and *P. phymatum* have wide distribution in both the invaded and the original regions of the *Mimosa* species and *P. caribensis* (Bontemps et al., 2010; dos Reis et al., 2010; Chen et al., 2005a, b, 2006; Elliott et al., 2007, 2009; Liu et al., 2011, 2012; Mishra et al., 2012; Gehlot et al., 2013; Lardi et al., 2017). In addtion, its greater abundance in Yunnan Province (Liu et al., 2011, 2012) than in neighbor provinces examined in the present study demonstrated that *P. mimosarum* strains were more adapted to the soil in Yunnan. *P. caribensis* was originally described for non-symbiotic strains isolated from soil (Achouak et al., 1999) and symbionts of *Mimosa* belonging to this species were isolated lately in China (Chen et al., 2003b; Liu et al., 2012). So, again, its possible that novel symbiont of *Mimosa* may have evolved in China after this plant was introduced.

*Rhizobium* was isolated as minor group from the *Mimosa* species in this study (Table 1), and most of them (12 of the 14 strains) were from Hainan Province, with few isolates from Yunnan (Liu et al., 2012) and Guangxi (Table 1). Based upon the MLSA results (Figure 2) these strains were idertified as *R. mesoamericanum*, *R. etli*, and *R. altiplani*, while all of them were identified as sv. mimosa according to the nodA and nifG phylogenies (Figure 3 and Supplementary Figure S6). Previously, *R. etli* sv. mimosae has isolated from invasive Mimosa species in low frequency (Chen et al., 2001, 2003b, 2005a; Elliott et al., 2009; Klonowska et al., 2012; Mishra et al., 2012; Melkonian et al., 2014), while both *R. etli* and *R. mesoamericanum* were found to be predominate in *Mimosa* symbionts in Mexico, the second largest center of *Mimosa* diversity (Wang et al., 1999; Bontemps et al., 2016). The exception is *R. altiplani*, which is relatively common in central Brazil (Baraúna et al., 2016; de Castro Pires et al., 2018), but has never previously been isolated from *Mimosa* in its invasive range regions. So, the *Mimosa*-nodulation Rhizobium species in China might be also introduced together with their hosts, but they are not so adapted to the conditions in the invasive regions.

In addtition to the species definition, great genetic diversity represented by the 94 protein patterns and 63 BOX-PCR patterns (Table 1) was revealed in this study among the Alpha- and Beta-rhizobial symbionts isolated from only two *Mimosa* species. This might be related to the vast area of the sampling locations, which covered diverse soil types and forced the diversification of rhizobia for their survival.

In summary, five beta-rhizobial species, especially *Cupriavidus* genotype II, with great genetic diversity as the dominant microsymbionts and three *Rhizobium* species as minor microsymbionts for *M. pudica* and *M. diplotricha* plants were detected in a vast sampling area in China. Most of them have been found in both the invasive regions and the centers of origin of these plants, but *Cupriavidus* genotype II might be a novel symbiont for *Mimosa* species evolved in China.

### Soil Variables and the Distribution of Rhizobial Genotypes Associated With *Mimosa* Species

The worldwide distribution of various symbionts isolated from invasive *Mimosa* species may be the result of selection by soil characteristics and other ecological factors (Elliott et al., 2009; Melkonian et al., 2014; de Castro Pires et al., 2018). In the present study, the community composition in the five provinces were unbalanced: *P. mimosarum* was more abundant in Yunnan; *P. phymatum* was more in Guangdong and Guangxi; *Rhizobium* stains were mostly from Hainan; and the minor group *P. caribensis* was only isolated from *M. pudica* plants grown in Guangdong and Guangxi. These geographic distributions implied an interaction among the plants, the rhizobial species, and the environment factors, as described for rhizobia associated with soybean (Zhang et al., 2011).

In the SPSS analysis and the principal component analysi (Figure 4), the correlation of *Cupriavidus* spp. and *Rhizobium* spp. with the infertile alkaline soil type (category I) and neutral soil (category II), and the accommodating of *Paraburkholderia* spp. in more acidic soils (category III and IV) were consistent with the previous reports that have shown that *C. taiwanensis* is abundant as a *Mimosa* symbiont in neutral to basic pH soils (Table 2), often with relatively high fertility (Elliott et al., 2009; Klonowska et al., 2012; Mishra et al., 2012; Gehlot et al., 2013). In contrast to *Cupriavidus*, *Paraburkholderia* strains can tolerate acidic soils (Stopnisek et al., 2014), and indeed become dominant symbionts with the compatible legumes; it was similar for rhizobia nodulating with mimosoid and papilionoid legumes (Garau et al., 2009; dos Reis et al., 2010; Howieson et al., 2013; Liu et al., 2014; Lemaire et al., 2015, 2016a,b). The present study has clearly illustrated that pH appears to be the most important environmental factor in helping to explain the distribution of *Mimosa* symbionts in southern China (Table 2, *p* = 0.001, ≤0.01). For example, although higher soil fertility and N concentration improved the competitive nodulation of *Paraburkholderia* over *Cupriavidus* and *Rhizobium* on *Mimosa* spp. in reduced N concentrations/low fertility growth media (Elliott et al., 2009), this was not the case in the present study wherein the dominant symbionts in the alkaline-neutral soils (soil types I and II) were overwhelmingly *Cupriavidus* regardless of their low fertility. The opposite was the case for the acidic soils wherein the *Paraburkholderia* strains dominated even though the soils were relatively fertile.

Taken together, *Cupriavidus* spp. are the most common and competitive rhizobial type in southern China due to its ability to grow in the widest range of pH, soil nutrition/fertility, and soil moisture levels (from drought to flooding), and maybe also to its tolerance to heavy metals (Klonowska et al., 2012). *C. taiwanensis* was recognized as strong stress resistant bacteria able to survive and grow at phenol concentrations up to 900 mg/L (Chen et al., 2004). It is also particularly dominant in islands like Taiwan (Chen et al., 2003b), New Caledonia (Klonowska et al., 2012), and Hainan (Table 1). Certainly it is possible that the similar climate and soils in these islands have created habitats ideal for the two invasive *Mimosa* species (*M. pudica*, *M. diplotricha*), where the soils are also rich in P and K, and would favor *C. taiwanensis* rather than (for instance) *Rhizobium*.

On the other hand, the dominance of *Cupriavidus* as a *Mimosa* symbionts appears to be a phenomenon that occurs mainly in invasive ecosystems. For example, in some natural ecosystems, such as central Mexico (Bontemps et al., 2016), the Indian Thar Desert (Gehlot et al., 2013), and even in parts of central Brazil (Baraúna et al., 2016; de Castro Pires et al., 2018), where soils are neutral to alkaline, Alpha-rhizobia can be the dominant symbionts of the native/endemic *Mimosa* species. Indeed, *Cupriavidus* species are normally minor group or absent in their centers of origin (Bontemps et al., 2010; dos Reis et al., 2010), de Castro Pires et al., 2018). The exceptions come from Texas where a widespread species *M. asperata* was nodulated only with *C. taiwanensis*-like bacteria (Andam et al., 2007), and from Uruguay in which native/endemic *Mimosa* species grown in slightly acidic soils of a heavy metal mining area were exclusively nodulated with *C. necator*- and *C. pinatubonensis*-like bacteria with *nod* genes divergent from *C. taiwanensis* (Platero et al., 2016). Interestingly, the Uruguayan native/endemic *Mimosa* appeared to be incapable of nodulating effectively with *Paraburkholderia* strains suggesting that they had co-evolved with their *Cupriavidus* symbionts in a manner similar to that described for species in central Brazil and central Mexico with *Paraburkholderia* and Alpha-rhizobia symbionts, respectively (Bontemps et al., 2010, 2016).

In summary, *Cupriavidus* strains are highly adaptable and competitive symbionts of the two *Mimosa* species in an invasive context, particularly when soils are neutral-alkaline, but they even retain a high degree of competitiveness in soils less optimal for it (i.e., slightly acidic and with low levels of OM and N). The disparity between the dominance of *Cupariavidus* in an invasive environment, and its sporadic occurrence in their native regions is still not clearly explained, but it could be related to the fact that most *Mimosa* species are not capable of nodulating effectively with *C. taiwanensis* (Elliott et al., 2007; dos Reis et al., 2010). Interestingly, its preferred hosts, *M. pudica* and *M. diplotricha*, although common in lowland areas of the neotropics (Barrett and Parker, 2005, 2006; Mishra et al., 2012), are mainly restricted to disturbed sites (Bontemps et al., 2010; Baraúna et al., 2016). Therefore, it is possible that the same soil factors which encouraging the invasiveness of its *Mimosa* plants (fertile ground subject to anthropogenic disturbance) also created a niche which favoring *C. taiwanensis* over its usual competitors for nodulation in its native region (i.e., *Paraburkholderia* species).

## Data Availability Statement

The datasets presented in this study can be found in online repositories. The names of the repository/repositories and accession number(s) can be found in the Supplementary Material, Supplementary Table S2.

## Author Contributions

XYL conceived and designed the study. SHY, BJY, HYW, and FW collected the nodules from *Mimosa* and isolated the rhizobia strains. WDC and ZKL performed the soil nutrients detection, HJL conduct the PCA analysis, XYL and EKJ wrote the manuscript and edited the manuscript. All authors read and approved the manuscript.

## Conflict of Interest

The authors declare that the research was conducted in the absence of any commercial or financial relationships that could be construed as a potential conflict of interest.

## Supplementary Material

Supplementary Figure 1 Phylogenetic tree based on *recA* gene sequences (382 bp) showing the groups of the rhizobial strains isolated from *Mimosa* spp. The tree was constructed by using the Maximum likelihood method. Bootstrap values are indicated (>50) in the main nodes in a bootstrap analysis of 1,000 replicates, 5% substitutions per site.

Supplementary Figure 2 Phylogenetic tree based on *gyrB* gene sequences (432 bp) showing the groups of the rhizobial strains isolated from *Mimosa* spp. The tree was constructed by using the Maximum likelihood method. Bootstrap values are indicated (>50) in the main nodes in a bootstrap analysis of 1,000 replicates, 10% substitutions per site.

Supplementary Figure 3 Phylogenetic tree based on *atpD* gene sequences (434 bp) showing the groups ofthe rhizobial strains isolated from *Mimosa* spp. The tree was constructed by using the Maximum likelihood method. Bootstrap values are indicated (>50) in the main nodes in a bootstrap analysis of 1,000 replicates, 5% substitutions per site.

Supplementary Figure 4 Phylogenetic tree base on *dnaK* gene sequences (260 bp) showing the groups of the rhizobial strains isolated from *Mimosa* spp. The tree was constructed by using the Maximum likelihood method. Bootstrap values are indicated (>50) in the main nodes in a bootstrap analysis of 1,000 replicates, 10% substitutions per site.

Supplementary Figure 5 Phylogenetic tree based on *glnA* gene sequences (874 bp) showing the groups of the rhizobial strains isolated from *Mimosa* spp. The tree was constructed by using Maximum likelihood method. Bootstrap values are indicated (>50) in the main nodes in a bootstrap analysis of 1,000 replicates, 20% substitutions per site.

Supplementary Figure 6 Phylogenetic tree based on *nifH* gene sequences (253 bp) showing the groups of the rhizobial strains isolated from *Mimosa* spp. The tree was constructed by using the Maximum likelihood method. Bootstrap values are indicated (>50) in the main nodes in a bootstrap analysis of 1,000 replicates, 5% substitutions per site.

## References

[B1] Achouak W. Christen R. Barakat M. Martel M.-H. Heulin T. (1999). *Burkholderia caribensis* sp. nov., an exopolysaccharide-producing bacterium isolated from vertisol microaggregates in Martinique. *Int. J. Syst. Bacteriol.* 49 787–794. 10.1099/00207713-49-2-787 10319504

[B2] Andam C. P. Mondo S. J. Parker M. A. (2007). Monophyly of *nodA* and *nifH* genes across Texan and Costa Rican populations of Cupriavidus nodule symbionts. *Appl. Environ. Microbiol.* 73 4686–4690. 10.1128/AEM.00160-07 17526782 PMC1932833

[B3] Andam C. P. Parker M. A. (2007). Novel alpha*proteobacteria*l root nodule symbiont associated with *Lupinus texensis*. *Appl. Environ. Microbiol.* 73 5687–5691. 10.1128/AEM.01413-07 17616612 PMC2042092

[B4] Andrews M. De Meyer S. James E. Stępkowski T. Hodge S. Simon M. (2018). Horizontal Transfer of Symbiosis Genes within and Between Rhizobial Genera: occurrence and Importance. *Genes* 9:321. 10.3390/genes9070321 29954096 PMC6071183

[B5] Andrus A. D. Andam C. Parker M. A. (2012). American origin of *Cupriavidus* bacteria associated with invasive *Mimosa* legumes in the Philippines. *FEMS Microbiol. Ecol.* 80 747–750. 10.1111/j.1574-6941.2012.01342.x 22381032

[B6] Bamba M. Aoki S. Kajita T. Setoguchi H. Watano Y. Sato S. (2019). Exploring genetic diversity and signatures of horizontal gene transfer in nodule bacteria associated with lotus japonicus in natural environments. *Mol. Plant Microbe Interact.* 32 1110–1120. 10.1094/MPMI-02-19-0039-R 30880586

[B7] Baraúna A. C. Rouws L. F. M. Simoes-Araujo J. L. dos Reis Junior F. B. Iannetta P. P. M. Maluk M. (2016). *Rhizobium altiplani* sp. nov., isolated from effective nodules on *Mimosa pudica* growing in untypically alkaline soil in central Brazil. *Int. J. Syst. Evol. Microbiol.* 66 4118–4124. 10.1099/ijsem.0.001322 27453319

[B8] Barrett C. F. Parker M. A. (2005). Prevalence of *Burkholderia* sp. nodule symbionts on four mimosoid legumes from Barro Colorado Island, Panama. *Syst. Appl. Microbiol.* 28 57–65. 10.1016/j.syapm.2004.09.002 15709366

[B9] Barrett C. F. Parker M. A. (2006). Coexistence of *Burkholderia*, *Cupriavidus*, and *Rhizobium* sp. nodule bacteria on two *Mimosa* spp. in Costa Rica. *Society* 72 1198–1206. 10.1128/AEM.72.2.1198-1206.2006 16461667 PMC1392967

[B10] Beukes C. W. Palmer M. Manyaka P. Chan W. Y. Avontuur J. R. van Zyl E. (2017). Genome data provides high support for generic boundaries in *Burkholderia* sensu lato. *Front. Microbiol.* 8, 1–11. 10.3389/fmicb.2017.01154 28694797 PMC5483467

[B11] Bontemps C. Elliott G. N. Simon M. F. Dos Reis Júnior F. B. Gross E. Lawton R. C. (2010). *Burkholderia* species are ancient symbionts of legumes. *Mol. Ecol.* 19 44–52. 10.1111/j.1365-294X.2009.04458.x 20002602

[B12] Bontemps C. Rogel M. A. Wiechmann A. Mussabekova A. Moody S. Simon M. F. (2016). Endemic *Mimosa* species from Mexico prefer alpha*proteobacteria*l rhizobial symbionts. *New Phytol.* 209 319–333. 10.1111/nph.13573 26214613

[B13] Bournaud C. de Faria S. M. dos Santos J. M. F. Tisseyre P. Silva M. Chaintreuil C. (2013). *Burkholderia* Species Are the Most Common and Preferred Nodulating Symbionts of the *Piptadenia* Group (Tribe Mimoseae). *PLoS One* 8:e63478. 10.1371/journal.pone.0063478 23691052 PMC3655174

[B14] Chen W. M. Chang J. S. Wu C. H. Chang S. C. (2004). Characterization of phenol and trichloroethene degradation by the rhizobium *Ralstonia taiwanensis*. *Res. Microbiol.* 155 672–680. 10.1016/j.resmic.2004.05.0045 15380556

[B15] Chen W. M. de Faria S. M. Straliotto R. Pitard R. M. Simões-Araùjo J. L. Chou J. (2005a). Proof that *Burkholderia* Strains Form Effective Symbioses with Legumes: a Study of Novel *Mimosa*-Nodulating Strains from South America. *Appl. Environ. Microbiol.* 71 7461–7471. 10.1128/AEM.71.11.7461-7471.2005 16269788 PMC1287612

[B16] Chen W.-M. James E. K. Chou J.-H. Sheu S.-Y. Yang S.-Z. Sprent J. I. (2005b). Beta-Rhizobia from *Mimosa pigra*, a newly discovered invasive plant in Taiwan. *New Phytol.* 168 661–675. 10.1111/j.1469-8137.2005.01533.x 16313648

[B17] Chen W. M. James E. K. Coenye T. Chou J. H. Barrios E. de Faria S. M. (2006). *Burkholderia mimosarum* sp. nov., isolated from root nodules of *Mimosa* spp. from Taiwan and South America. *Int. J. Syst. Evol. Microbiol.* 56 1847–1851. 10.1099/ijs.0.64325-0 16902019

[B18] Chen W. M. James E. K. Prescott A. R. Kierans M. Sprent J. I. (2003a). Nodulation of *Mimosa* spp. by the beta-proteobacterium *Ralstonia taiwanensis*. *Mol. Plant Microbe Interact.* 16 1051–1061. 10.1094/MPMI.2003.16.12.1051 14651338

[B19] Chen W. M. Laevens S. Lee T. M. Coenye T. De Vos P. Mergeay M. (2001). *Ralstonia taiwanensis* sp. nov., isolated from root nodules of *Mimosa* species and sputum of a cystic fibrosis patient. *Int. J. Syst. Evol. Microbiol.* 51 1729–1735. 10.1099/00207713-51-5-1729 11594603

[B20] Chen W.-M. Moulin L. Bontemps C. Vandamme P. Béna G. Boivin-Masson C. (2003b). Legume symbiotic nitrogen fixation by beta-*proteobacteria* is widespread in nature. *J. Bacteriol.* 185 7266–7272. 10.1128/JB.185.24.7266-7272.2003 14645288 PMC296247

[B21] da Silva K. Florentino L. A. da Silva K. B. de Brandt E. Vandamme P. de Souza Moreira F. M. (2012). *Cupriavidus necator* isolates are able to fix nitrogen in symbiosis with different legume species. *Syst. Appl. Microbiol.* 35 175–182. 10.1016/j.syapm.2011.10.005 22361568

[B22] de Castro Pires R. dos Reis Junior F. B. Zilli J. E. Fischer D. Hofmann A. James E. K. (2018). Soil characteristics determine the rhizobia in association with different species of *Mimosa* in central Brazil. *Plant Soil* 423 411–428. 10.1007/s11104-017-3521-5

[B23] dos Reis F. B. Simon M. F. Gross E. Boddey R. M. Elliott G. N. Neto N. E. (2010). Nodulation and nitrogen fixation by *Mimosa* spp. in the Cerrado and Caatinga biomes of Brazil. *New Phytol.* 186 934–946. 10.1111/j.1469-8137.2010.03267.x 20456044

[B24] Du S. Gao X. (2006). *Technical Specification of Soil Analysis.* Beijing: China Agriculture Press.

[B25] Elliott G. N. Chen W. M. Chou J. H. Wang H. C. Sheu S. Y. Perin L. (2007). *Burkholderia phymatum* is a highly effective nitrogen-fixing symbiont of *Mimosa* spp. and fixes nitrogen ex planta. *New Phytol.* 173 168–180. 10.1111/j.1469-8137.2006.01894.x 17176403

[B26] Elliott G. N. Chou J. H. Chen W. M. Bloemberg G. V. Bontemps C. Martínez-Romero E. (2009). *Burkholderia* spp. are the most competitive symbionts of *Mimosa*, particularly under N-limited conditions. *Environ. Microbiol.* 11 762–778. 10.1111/j.1462-2920.2008.01799.x 19040456

[B27] Garau G. Yates R. J. Deiana P. Howieson J. G. (2009). Novel strains of nodulating *Burkholderia* have a role in nitrogen fixation with papilionoid herbaceous legumes adapted to acid, infertile soils. *Soil Biol. Biochem.* 41 125–134. 10.1016/j.soilbio.2008.10.011

[B28] Gehlot H. S. Tak N. Kaushik M. Mitra S. Chen W. M. Poweleit N. (2013). An invasive *Mimosa* in India does not adopt the symbionts of its native relatives. *Ann. Bot.* 112 179–196. 10.1093/aob/mct112 23712450 PMC3690997

[B29] Guan Z. B. Deng W. H. Huang Z. L. Huang N. Y. Ai L. Li C. (2006). A preliminary investigation on the alien invasive plants in Xishuangbanna. *Trop. Agric. Sci. Technol.* 29 35–38.

[B30] Gyaneshwar P. Hirsch A. M. Moulin L. Chen W.-M. Elliott G. N. Bontemps C. (2011). Legume-Nodulating Beta*proteobacteria*: diversity, host range, and future prospects. *Mol. Plant Microbe Interact.* 24 1276–1288. 10.1094/MPMI-06-11-0172 21830951

[B31] Haukka K. Lindström K. Young J. (1998). Three phylogenetic groups of *nodA* and *nifH* genes in Sinorhizobium and Mesorhizobium isolates from leguminous trees growing in Africa and Latin America. *Appl. Environ. Microbiol.* 64 419–426. 10.1128/AEM.64.2.419-426.1998 9464375 PMC106060

[B32] Howieson J. G. De Meyer S. E. Vivas-Marfisi A. Ratnayake S. Ardley J. K. Yates R. J. (2013). Novel *Burkholderia* bacteria isolated from *Lebeckia ambigua* - A perennial suffrutescent legume of the fynbos. *Soil Biol. Biochem.* 60 55–64. 10.1016/j.soilbio.2013.01.009

[B33] Huo Y. Tong W. Wang J. Wang F. Bai W. Wang E. (2019). *Rhizobium chutanense* sp. nov., isolated from root nodules of *phaseolus vulgaris* in China. *Int. J. Syst. Evol. Microbiol.* 69 2049–2056. 10.1099/ijsem.0.003430 31091180

[B34] Klonowska A. Chaintreuil C. Tisseyre P. Miché L. Melkonian R. Ducousso M. (2012). Biodiversity of **Mimosa* pudica* rhizobial symbionts (*Cupriavidus taiwanensis*, *Rhizobium mesoamericanum*) in New Caledonia and their adaptation to heavy metal-rich soils. *FEMS Microbiol. Ecol.* 81 618–635. 10.1111/j.1574-6941.2012.01393.x 22512707

[B35] Laemmli U. K. (1970). Cleavage of structural proteins during the assembly of the head of bacteriophage T4. *Nature* 227 680–685. 10.1038/227680a0 5432063

[B36] Laguerre G. Allard M.-R. Revoy F. Amarger N. (1994). Rapid Identification of Rhizobia by Restriction Fragment Length Polymorphism Analysis of PCR-Amplified 16S rRNA Genes. *Appl. Environ. Microbiol.* 60 56–63. 10.1128/AEM.60.1.56-63.1994 16349165 PMC201269

[B37] Laguerre G. Nour S. M. Macheret V. Sanjuan J. Drouin P. Amarger N. (2001). Classification of rhizobia based on *nodC* and *nifH* gene analysis reveals a close phylogenetic relationship among *Phaseolus vulgaris* symbionts. *Microbiology* 147 981–993. 10.1099/00221287-147-4-981 11283294

[B38] Lardi M. de Campos S. B. Purtschert G. Eberl L. Pessi G. (2017). Competition experiments for legume infection identify *Burkholderia phymatum* as a highly competitive β-rhizobium. *Front. Microbiol.* 8:1527. 10.3389/fmicb.2017.01527 28861050 PMC5559654

[B39] Lemaire B. Chimphango S. B. M. Stirton C. Rafudeen S. Honnay O. Smets E. (2016a). Biogeographical patterns of legume-nodulating *Burkholderia* spp.: from African fynbos to continental scales. *Appl. Environ. Microbiol.* 82 5099–5115. 10.1128/AEM.00591-16 27316955 PMC4988186

[B40] Lemaire B. Dlodlo O. Chimphango S. Stirton C. Schrire B. Boatwright J. S. (2015). Symbiotic diversity, specificity and distribution of rhizobia in native legumes of the Core Cape Subregion (South Africa). *FEMS Microbiol. Ecol.* 91 1–17. 10.1093/femsec/fiu024 25764552

[B41] Lemaire B. Van Cauwenberghe J. Verstraete B. Chimphango S. Stirton C. Honnay O. (2016b). Characterization of the papilionoid-*Burkholderia* interaction in the Fynbos biome: the diversity and distribution of beta-rhizobia nodulating Podalyria calyptrata (Fabaceae, Podalyrieae). *Syst. Appl. Microbiol.* 39 41–48. 10.1016/j.syapm.2015.09.006 26689612

[B42] Li Y. H. Wang R. Sui X. H. Wang E. T. Zhang X. X. Tian C. F. (2019). *Bradyrhizobium nanningense* sp. nov., *Bradyrhizobium guangzhouense* sp. nov. and *Bradyrhizobium zhanjiangense* sp. nov., isolated from effective nodules of peanut in Southeast China. *Syst. Appl. Microbiol.* 42:126002. 10.1016/j.syapm.2019.126002 31362902

[B43] Liu W. Y. Y. Ridgway H. J. James T. K. James E. K. Chen W. M. Sprent J. I. (2014). *Burkholderia* sp. Induces Functional Nodules on the South African Invasive Legume *Dipogon lignosus* (Phaseoleae) in New Zealand Soils. *Microb. Ecol.* 68 542–555. 10.1007/s00248-014-0427-0 24801964

[B44] Liu X. Y. Wei S. Wang F. James E. K. Guo X. Zagar C. (2012). *Burkholderia* and *Cupriavidus* spp. are the preferred symbionts of **Mimosa** spp. in Southern China. *FEMS Microbiol. Ecol.* 80 417–426. 10.1111/j.1574-6941.2012.01310.x 22268711

[B45] Liu X. Y. Wang E. T. Li Y. Chen W. X. (2007). Diverse bacteria isolated from root nodules of *Trifolium*, *Crotalaria* and **Mimosa** grown in the subtropical regions of China. *Arch. Microbiol.* 188 1–14. 10.1007/s00203-007-0209-x 17497134

[B46] Liu X. Y. Wu W. Wang E. T. Zhang B. Macdermott J. Chen W. X. (2011). Phylogenetic relationships and diversity of β-rhizobia associated with *Mimosa* species grown in Sishuangbanna, China. *Int. J. Syst. Evol. Microbiol.* 61 334–342. 10.1099/ijs.0.020560-0 20228206

[B47] Los Santos P. Palmer M. Beukes C. Steenkamp E. T. Briscoe L. Id N. K. (2018). Whole Genome Analyses Suggests that *Burkholderia* sensu lato Contains Two Additional Novel Genera Implications for the Evolution of Diazotrophy and Nodulation in the *Burkholderiaceae*. *Gene* 9 1–23. 10.3390/genes9080389 30071618 PMC6116057

[B48] Martens M. Dawyndt P. Coopman R. Gillis M. De Vos P. Willems A. (2008). Advantages of multilocus sequence analysis for taxonomic studies: a case study using 10 housekeeping genes in the genus Ensifer (including former Sinorhizobium). *Int. J. Syst. Evol. Microbiol.* 58 200–214. 10.1099/ijs.0.65392-0 18175710

[B49] Martens M. Delaere M. Coopman R. De Vos P. Gillis M. Willems A. (2007). Multilocus sequence analysis of *Ensifer* and related taxa. *Int. J. Syst. Evol. Microbiol.* 57 489–503. 10.1099/ijs.0.64344-0 17329774

[B50] Melkonian R. Moulin L. Béna G. Tisseyre P. Chaintreuil C. Heulin K. (2014). The geographical patterns of symbiont diversity in the invasive legume **Mimosa* pudica* can be explained by the competitiveness of its symbionts and by the host genotype. *Environ. Microbiol.* 16 2099–2111. 10.1111/1462-2920.12286 24131520

[B51] Mishra R. P. N. Tisseyre P. Melkonian R. Chaintreuil C. Miché L. Klonowska A. (2012). Genetic diversity of *Mimosa pudica* rhizobial symbionts in soils of French Guiana: investigating the origin and diversity of *Burkholderia phymatum* and other beta-rhizobia. *FEMS Microbiol. Ecol.* 79 487–503. 10.1111/j.1574-6941.2011.01235.x 22093060

[B52] Nick G. de Lajudie P. Eardly B. D. Suomalainen S. Paulin L. Zhang X. P. (1999). *Sinorhizobium arboris* sp nov and *Sinorhizobium kostiense* sp nov., isolated from leguminous trees in Sudan and Kenya. *Int. J. Syst. Bacteriol.* 49 1359–1368. 10.1099/00207713-49-4-1359 10555313

[B53] Parker M. A. Wurtz A. K. Paynter Q. (2007). Nodule symbiosis of invasive *Mimosa pigra* in Australia and in ancestral habitats: a comparative analysis. *Biol. Invasions* 9 127–138. 10.1007/s10530-006-0009-2

[B54] Payne G. W. Vandamme P. Morgan S. H. LiPuma J. J. Coenye T. Weightman A. J. (2005). Development of a recA Gene-Based Identification Approach for the Entire *Burkholderia* Genus. *Appl. Environ. Microbiol.* 21 3917–3927. 10.1128/AEM.71.7.3917-3927.2005 16000805 PMC1169057

[B55] Peix A. Ramírez-bahena M. H. Velázquez E. Eulogio J. Peix A. Ram M. H. (2015). Critical reviews in plant sciences bacterial associations with legumes bacterial associations with legumes. *Crit. Rev. Plant Sci.* 34 17–42. 10.1080/07352689.2014.897899

[B56] Platero R. James E. K. Rios C. Iriarte A. Sandes L. Zabaleta M. (2016). Novel *Cupriavidus* strains isolated from root nodules of native Uruguayan *Mimosa* species. *Appl. Environ. Microbiol.* 82 3150–3164. 10.1128/AEM.04142-15 26994087 PMC4959248

[B57] Silva V. C. Alves P. A. C. Rhem M. F. K. dos Santos J. M. F. James E. K. Gross E. (2018). Brazilian species of *Calliandra* Benth. (tribe Ingeae) are nodulated by diverse strains of Paraburkholderia. *Syst. Appl. Microbiol.* 41 241–250. 10.1016/j.syapm.2017.12.003 29336852

[B58] Sneath P. H. A. Sokal R. R. (1973). *Numerial Taxonomy. The Principles and Practice of Classification.* San Francisco, CA: W. H. Freeman.

[B59] Sprent J. (2009). *Legume Nodulation: A Global Perspective*, 1st Edn Hoboken, NJ: Wiley 10.1002/9781444316384

[B60] Sprent J. I. Ardley J. James E. K. (2017). Biogeography of nodulated legumes and their nitrogen-fixing symbionts. *New Phytol.* 215 40–56. 10.1111/nph.14474 28211601

[B61] Stopnisek N. Bodenhausen N. Frey B. Fierer N. Eberl L. Weisskopf L. (2014). Genus-wide acid tolerance accounts for the biogeographical distribution of soil *Burkholderia* populations. *Environ. Microbiol.* 16 1503–1512. 10.1111/1462-2920.12211 23945027

[B62] Sun L. N. Wang D. S. Yang E. D. Fang L. C. Chen Y. F. Tang X. Y. (2016). *Cupriavidus nantongensis* sp. nov., a novel chlorpyrifos-degrading bacterium isolated from sludge. *Int. J. Syst. Evol. Microbiol.* 66 2335–2341. 10.1099/ijsem.0.001034 27001671

[B63] Tamura K. Stecher G. Peterson D. Filipski A. Kumar S. (2013). MEGA6: molecular evolutionary genetics analysis version 6.0. *Mol. Biol. Evol.* 30 2725–2729. 10.1093/molbev/mst197 24132122 PMC3840312

[B64] Tan Z.-Y. Xu X.-D. Wang E.-T. Gao J.-L. Martinez-Romer E. Chen W.-X. (1997). Phylogenetic and Genetic Relationships of *Mesorhizobium tianshanense* and Related Rhizobia. *Int. J. Syst. Bacteriol.* 874–879. 10.1099/00207713-47-3-874 9226921

[B65] Taulé C. Zabaleta M. Mareque C. Platero R. Sanjurjo L. Sicardi M. (2012). New beta*proteobacteria*l Rhizobium strains able to efficiently nodulate *Parapiptadenia rigida* (Benth.) Brenan. *Appl. Environ. Microbiol.* 78 1692–1700. 10.1128/AEM.06215-11 22226956 PMC3298154

[B66] Thompson J. D. Gibson T. J. Plewniak F. Jeanmougin F. Higgins D. G. (1997). The CLUSTAL X windows interface: flexible strategies for multiple sequence alignment aided by quality analysis tools. *Nucleic Acids Res.* 25 4876–4882. 10.1093/nar/25.24.4876 9396791 PMC147148

[B67] Versalovic J. Koeuth T. Lupski J. R. (1991). Distribution of repetitive DNA sequences in eubacteria and application to fingerprinting of bacterial genomes. *Nucleic Acids Res.* 19 6823–6831. 10.1093/nar/19.24.6823 1762913 PMC329316

[B68] Vincent J, M. (1970). *A Manual for the Practical Study of the Root-Nodule Bacteria*. Oxford: Blackwell Scientific.

[B69] Vinuesa P. Silva C. Lorite M. J. Izaguirre-Mayoral M. L. Bedmar E. J. Martínez-Romero E. (2005). Molecular systematics of rhizobia based on maximum likelihood and Bayesian phylogenies inferred from *rrs, atpD, recA* and *nifH* sequences, and their use in the classification of *Sesbania* microsymbionts from Venezuelan wetlands. *Syst. Appl. Microbiol.* 28 702–716. 10.1016/j.syapm.2005.05.007 16261860

[B70] Wang E. T. Chen W. F. Tian C. F. Young J. P. W. Chen W. X. (2019). *Ecology and Evolution of Rhizobia: Principles and Applications.* Singapore: Springer Verlag 10.1007/978-981-32-9555-1

[B71] Wang E. T. Rogel M. A. Santos A. G. Martinez-romero J. Cevallos M. A. (1999a). *Rhizobium etli* bv. mimosae, a novel biovar isolated from *Mimosa* affinis. *Int. J. Syst. Bacteriol.* 49 1479–1491. 10.1099/00207713-49-4-1479 10555329

[B73] Wang G. Q. (2014). *Compendium of Chinese Traditional Herbal Drugs.* Beijing: People’s Health Press.

[B74] Weisburg W. G. Barns S. M. Pelletier D. A. Lane D. J. (1991). 16S ribosomal DNA amplification for phylogenetic study. *J. Bacteriol.* 173 697–703. 10.1128/JB.173.2.697-703.1991 1987160 PMC207061

[B75] Wu S. Chaw S. Rejmánek M. (2003). Naturalized Fabaceae (Leguminosae) in Taiwan: the first approximation. *Bot. Bull. Acad. Sin.* 44 59–66.

[B76] Zhang J. J. Guo C. Chen W. DeLajudie P. Zhang Z. Shang Y. (2018). *Mesorhizobium wenxiniae* sp. nov., isolated from chickpea (*Cicer arietinum* L.) in China. *Int. J. Syst. Bacteriol.* 68 1930–1936. 10.1099/ijsem.0.002770 29676730

[B77] Zhang J. J. Lou K. Jin X. Mao P. H. Wang E. T. Tian C. F. (2012). Distinctive Mesorhizobium populations associated with *Cicer arietinum* L. in alkaline soils of Xinjiang, China. *Plant Soil* 353 123–134. 10.1007/s11104-011-1014-5

[B78] Zhang J. J. Yang X. Guo C. de Lajudie P. Singh R. P. Wang E. (2017). *Mesorhizobium muleiense* and *Mesorhizobium* gsp. nov. are symbionts of *Cicer arietinum* L. in alkaline soils of Gansu, Northwest China. *Plant Soil* 410 103–112. 10.1007/s11104-016-2987-x

[B79] Zhang Y. M. Li Y. Chen W. F. Wang E. T. Tian C. F. Li Q. Q. (2011). Biodiversity and biogeography of rhizobia associated with soybean plants grown in the North China Plain. *Appl. Environ. Microbiol.* 77 6331–6342. 10.1128/AEM.00542-11 21784912 PMC3187167

