# Supplementary figures and images for: Diversity and Geographic Distribution of Microsymbionts Associated With Invasive Mimosa Species in Southern China
Source: Front Microbiol. 2020 Oct 28;11:563389. doi: 10.3389/fmicb.2020.563389 (PMC7673401; doi:10.3389/fmicb.2020.563389)

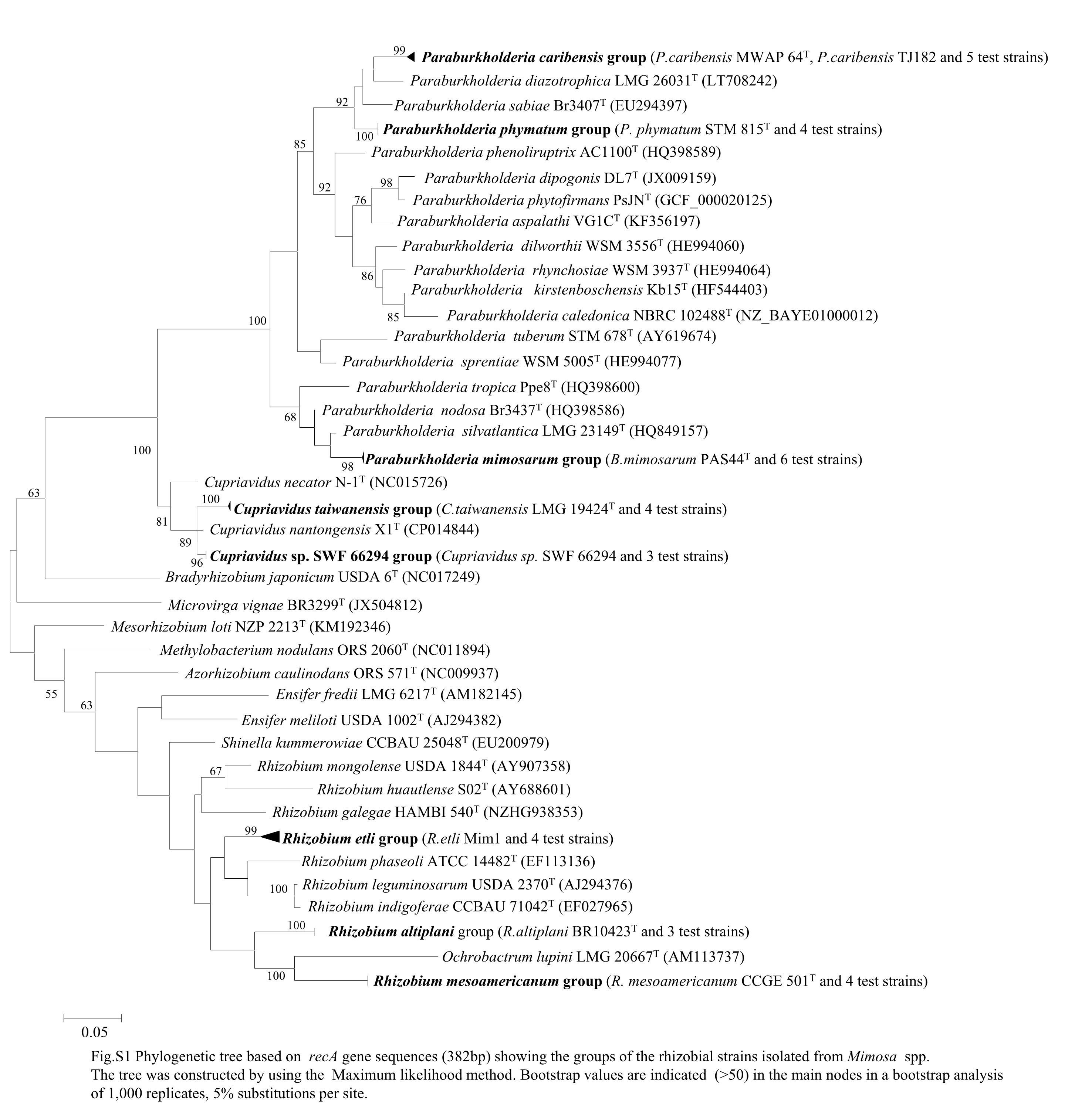

Supplement: Supplementary Figure 1 — Phylogenetic tree based on recA gene sequences (382 bp) showing the groups of the rhizobial strains isolated from Mimosa spp. The tree was constructed by using the Maximum likelihood method. Bootstrap values are indicated (>50) in the main nodes in a bootstrap analysis of 1,000 replicates, 5% substitutions per site. [file Image_1.JPEG]

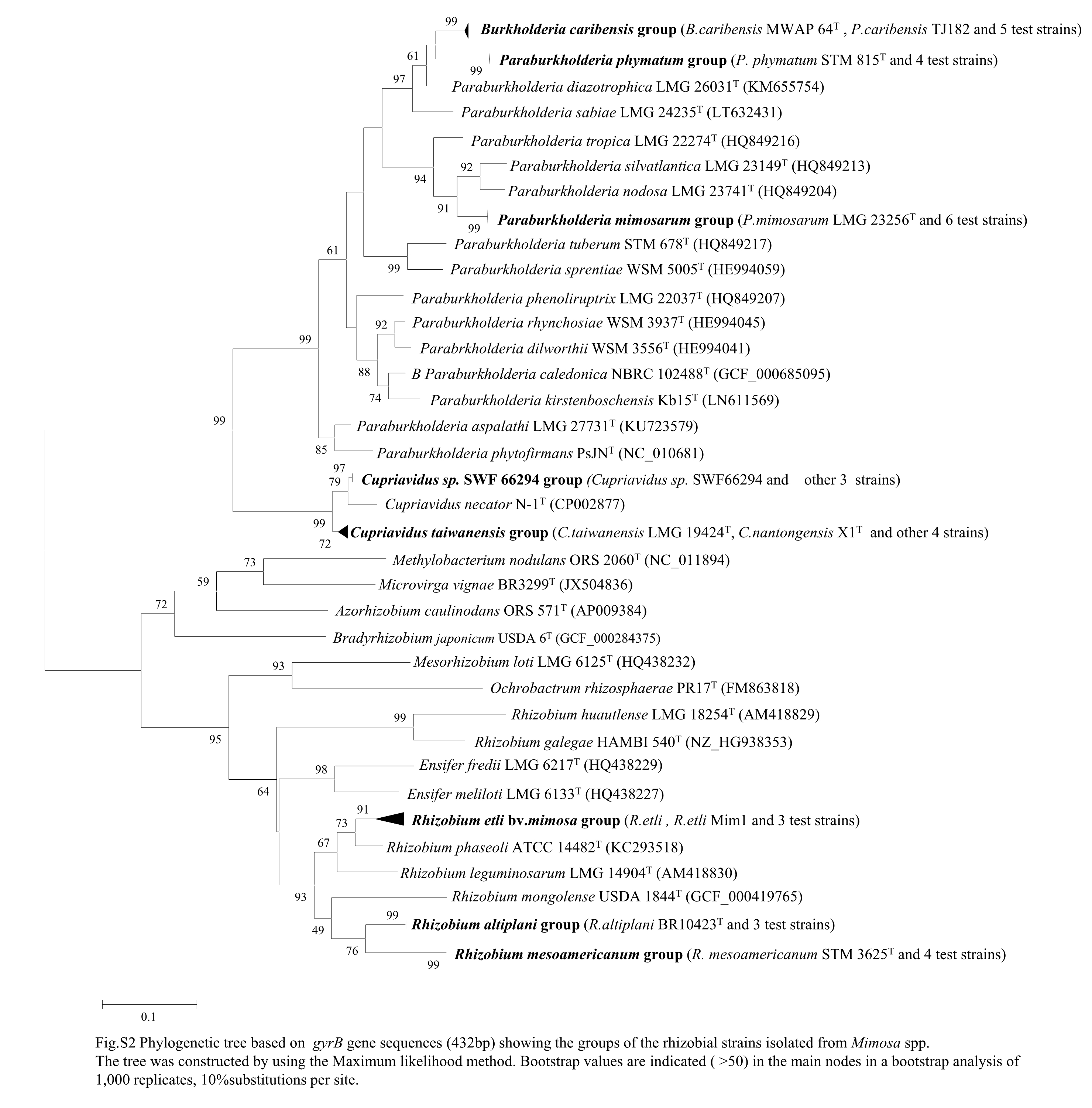

Supplement: Supplementary Figure 2 — Phylogenetic tree based on gyrB gene sequences (432 bp) showing the groups of the rhizobial strains isolated from Mimosa spp. The tree was constructed by using the Maximum likelihood method. Bootstrap values are indicated (>50) in the main nodes in a bootstrap analysis of 1,000 replicates, 10% substitutions per site. [file Image_2.JPEG]

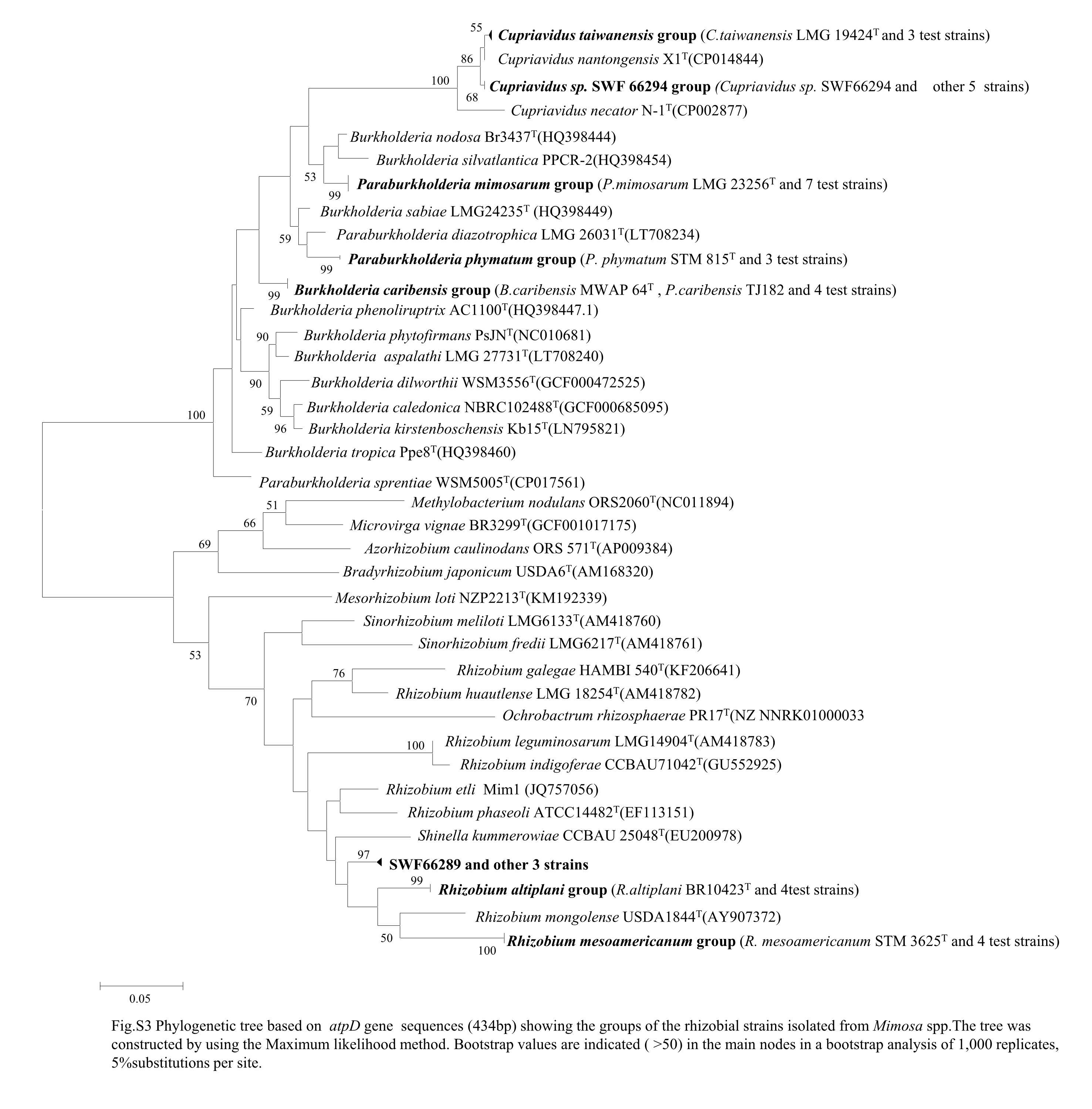

Supplement: Supplementary Figure 3 — Phylogenetic tree based on atpD gene sequences (434 bp) showing the groups ofthe rhizobial strains isolated from Mimosa spp. The tree was constructed by using the Maximum likelihood method. Bootstrap values are indicated (>50) in the main nodes in a bootstrap analysis of 1,000 replicates, 5% substitutions per site. [file Image_3.JPEG]

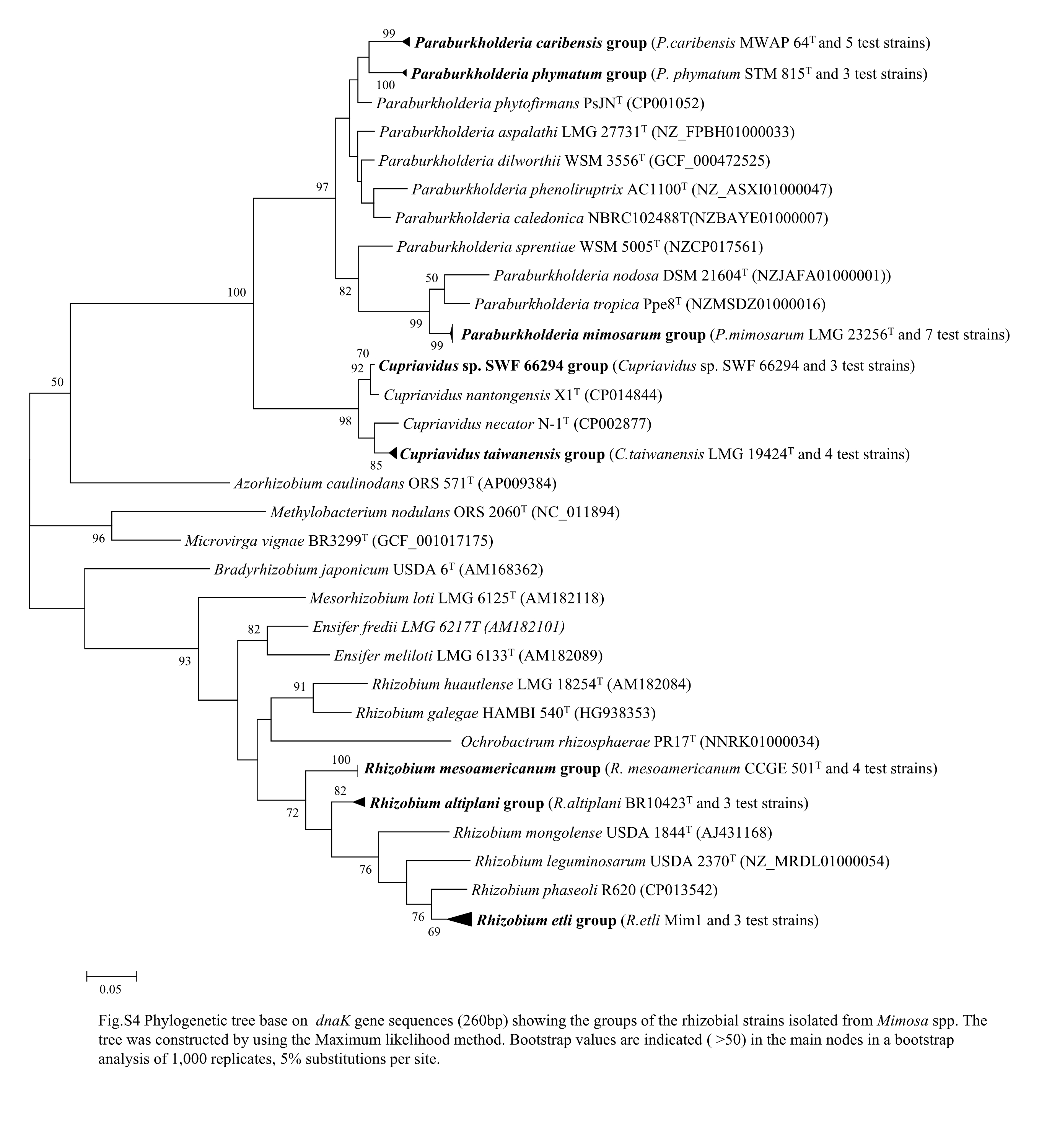

Supplement: Supplementary Figure 4 — Phylogenetic tree base on dnaK gene sequences (260 bp) showing the groups of the rhizobial strains isolated from Mimosa spp. The tree was constructed by using the Maximum likelihood method. Bootstrap values are indicated (>50) in the main nodes in a bootstrap analysis of 1,000 replicates, 10% substitutions per site. [file Image_4.JPEG]

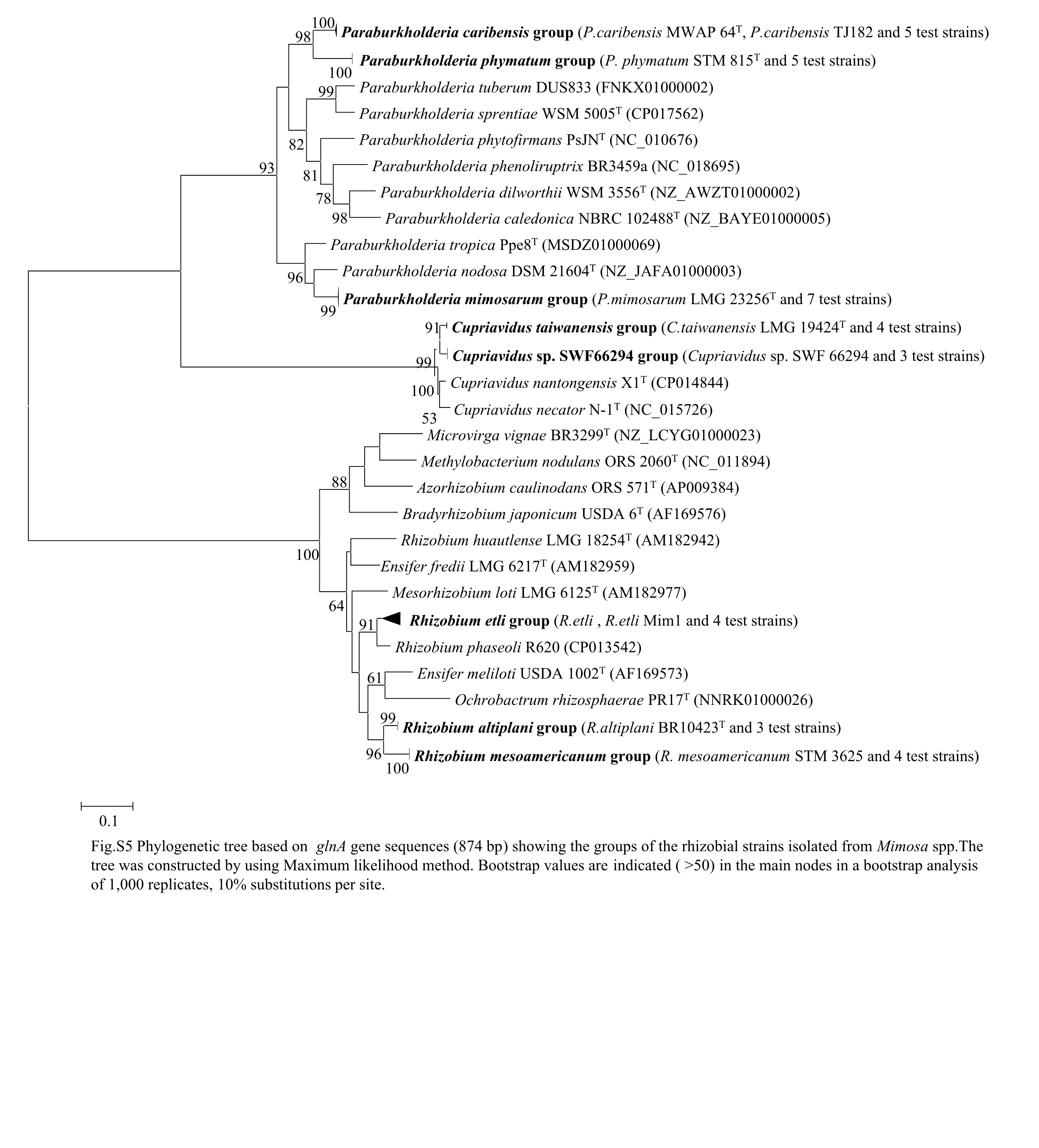

Supplement: Supplementary Figure 5 — Phylogenetic tree based on glnA gene sequences (874 bp) showing the groups of the rhizobial strains isolated from Mimosa spp. The tree was constructed by using Maximum likelihood method. Bootstrap values are indicated (>50) in the main nodes in a bootstrap analysis of 1,000 replicates, 20% substitutions per site. [file Image_5.JPEG]

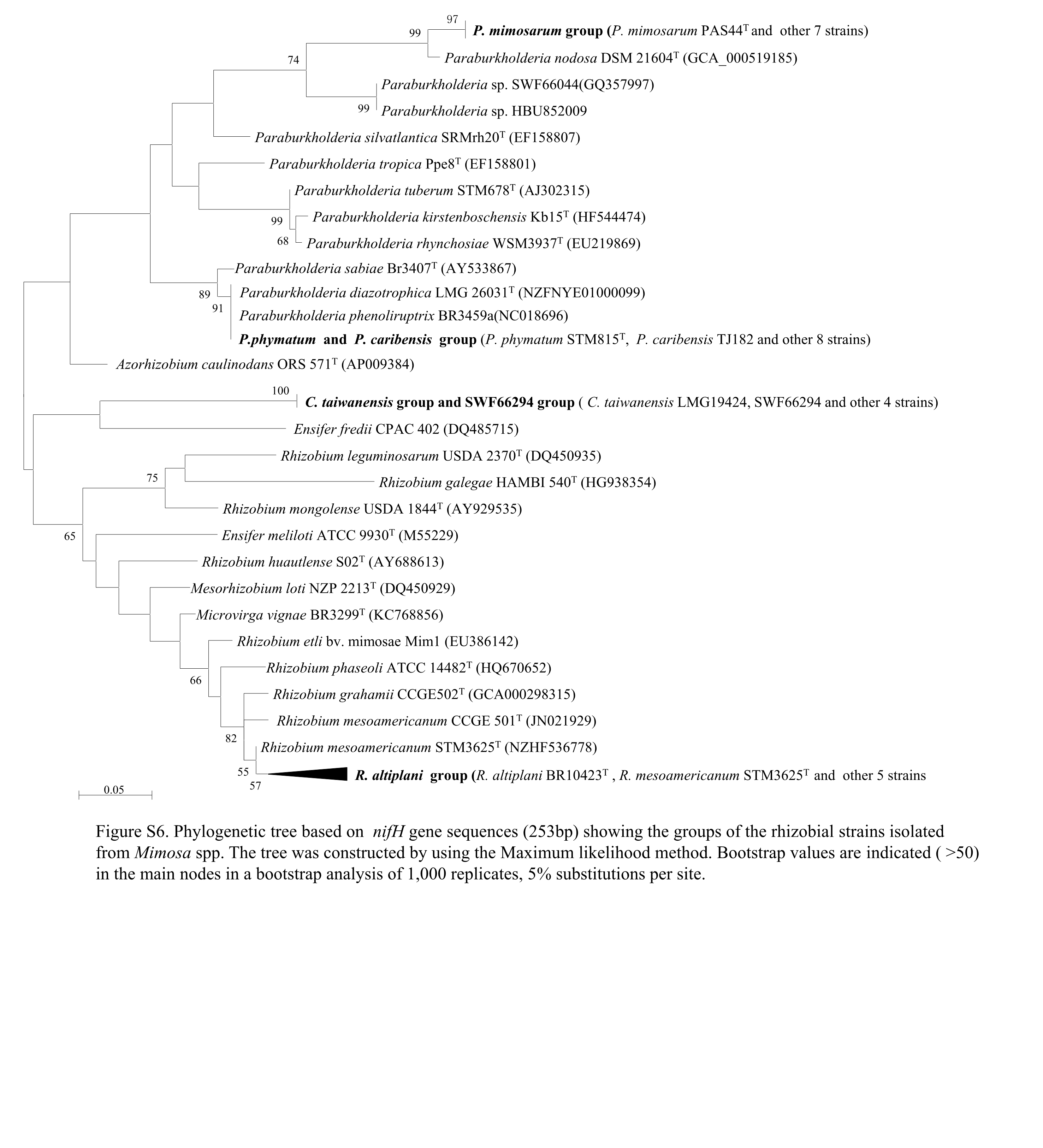

Supplement: Supplementary Figure 6 — Phylogenetic tree based on nifH gene sequences (253 bp) showing the groups of the rhizobial strains isolated from Mimosa spp. The tree was constructed by using the Maximum likelihood method. Bootstrap values are indicated (>50) in the main nodes in a bootstrap analysis of 1,000 replicates, 5% substitutions per site. [file Image_6.JPEG]
